# Mechanisms of distinctive mismatch tolerance between Rad51 and Dmc1 in homologous recombination

**DOI:** 10.1093/nar/gkab1141

**Published:** 2021-12-06

**Authors:** Jingfei Xu, Lingyun Zhao, Sijia Peng, Huiying Chu, Rui Liang, Meng Tian, Philip P Connell, Guohui Li, Chunlai Chen, Hong-Wei Wang

**Affiliations:** Ministry of Education Key Laboratory of Protein Sciences, Tsinghua-Peking Joint Center for Life Sciences, Beijing Advanced Innovation Center for Structural Biology, School of Life Sciences, Tsinghua University, Beijing, 100084, China; School of Life Sciences, Beijing Normal University, Beijing, 100875, China; Ministry of Education Key Laboratory of Protein Sciences, Tsinghua-Peking Joint Center for Life Sciences, Beijing Advanced Innovation Center for Structural Biology, School of Life Sciences, Tsinghua University, Beijing, 100084, China; Imaging and Characterization Core Lab, King Abdullah University of Science and Technology (KAUST), Thuwal, 23955-6900, Saudi Arabia; Beijing Advanced Innovation Center for Structural Biology, School of Life Sciences, Tsinghua University, Beijing, 100084, China; Laboratory of Molecular Modeling and Design, State Key Laboratory of Molecular Reaction Dynamics, Dalian Institute of Chemical Physics, Chinese Academy of Science, 457 Zhongshan Road, Dalian, 116023, China; Ministry of Education Key Laboratory of Protein Sciences, Tsinghua-Peking Joint Center for Life Sciences, Beijing Advanced Innovation Center for Structural Biology, School of Life Sciences, Tsinghua University, Beijing, 100084, China; Ministry of Education Key Laboratory of Protein Sciences, Tsinghua-Peking Joint Center for Life Sciences, Beijing Advanced Innovation Center for Structural Biology, School of Life Sciences, Tsinghua University, Beijing, 100084, China; Department of Radiation and Cellular Oncology, University of Chicago, Chicago, IL 60615, USA; Laboratory of Molecular Modeling and Design, State Key Laboratory of Molecular Reaction Dynamics, Dalian Institute of Chemical Physics, Chinese Academy of Science, 457 Zhongshan Road, Dalian, 116023, China; Beijing Advanced Innovation Center for Structural Biology, School of Life Sciences, Tsinghua University, Beijing, 100084, China; Ministry of Education Key Laboratory of Protein Sciences, Tsinghua-Peking Joint Center for Life Sciences, Beijing Advanced Innovation Center for Structural Biology, School of Life Sciences, Tsinghua University, Beijing, 100084, China

## Abstract

Homologous recombination (HR) is a primary DNA double-strand breaks (DSBs) repair mechanism. The recombinases Rad51 and Dmc1 are highly conserved in the RecA family; Rad51 is mainly responsible for DNA repair in somatic cells during mitosis while Dmc1 only works during meiosis in germ cells. This spatiotemporal difference is probably due to their distinctive mismatch tolerance during HR: Rad51 does not permit HR in the presence of mismatches, whereas Dmc1 can tolerate certain mismatches. Here, the cryo-EM structures of Rad51–DNA and Dmc1–DNA complexes revealed that the major conformational differences between these two proteins are located in their Loop2 regions, which contain invading single-stranded DNA (ssDNA) binding residues and double-stranded DNA (dsDNA) complementary strand binding residues, stabilizing ssDNA and dsDNA in presynaptic and postsynaptic complexes, respectively. By combining molecular dynamic simulation and single-molecule FRET assays, we identified that V273 and D274 in the Loop2 region of human RAD51 (hRAD51), corresponding to P274 and G275 of human DMC1 (hDMC1), are the key residues regulating mismatch tolerance during strand exchange in HR. This HR accuracy control mechanism provides mechanistic insights into the specific roles of Rad51 and Dmc1 in DNA double-strand break repair and may shed light on the regulatory mechanism of genetic recombination in mitosis and meiosis.

## INTRODUCTION

Eukaryotic recombinases Rad51 and Dmc1 belong to the RecA family and thus share many common properties (1). Rad51 and Dmc1 sequences are evolutionarily conserved, with Dmc1 about 45% identical to Rad51 in yeast and 54% identical to Rad51 in human (2–4). Both are ATPases and require ATP or its non-hydrolysable analogs to assemble on single-stranded DNA (ssDNA) as the presynaptic complex (5–8), which leads to searching for homologous double-stranded DNA (dsDNA) in chromosomes. After locating the homologous region, the presynaptic complex initiates strand invasion and exchange in a synaptic reaction process (9,10). Following strand exchange, the nascent homologous dsDNA wrapped by the protein is known as the postsynaptic complex, which also contains the displaced ssDNA product (11,12).

In eukaryotic cells, Rad51 is mostly responsible for homologous recombination (HR) between sister chromatids in somatic cells to ensure genome integrity (13), while Dmc1 functions specifically in germline cells during meiosis to recombine parental homologous chromatin for proper genetic inheritance and chromosome segmentation (14,15). Accumulating evidence has demonstrated that the mismatch tolerances of strand exchange processes mediated by these two recombinases are quite different (16,17). Comparing with the higher mismatch tolerance of Dmc1, strand exchange mediated by Rad51 aborts with only one or two mismatched bases. More recently, it was found that Rad51 is involved not only in mitosis for repairing a stalled replication fork but also in meiosis as an accessory factor for Dmc1 (18,19). The level of mismatch tolerance during HR is a critical parameter to be regulated in mitosis and meiosis pathways. Therefore, revealing the mechanism of distinctive mismatch tolerance between these two highly conserved proteins is essential to understanding the distinct HR processes for potential medical or biotechnical applications.

To understand the mechanisms of HR mediated by RecA family proteins, structures of different states of RecA, Rad51 and Dmc1 have been resolved in the past years. The structures of RecA–DNAs complexes reveal how neighboring RecA protomers interact with and stretch DNAs in both presynaptic and postsynaptic complexes (20). These interactions are highly conserved in hRAD51 (21,22). For Dmc1, octameric ring formation of hDMC1 has been resolved from crystallization of its full-length form in the absence of DNA (23). The most recent work has revealed the structures of hDMC1–DNAs complexes in both the presynaptic and postsynaptic states, which share similar structures as RecA–DNAs and hRAD51–DNAs complexes (24).

The dynamic strand exchange process mediated by RecA or Rad51 was further examined in detail by ensemble and single-molecule biochemical assays to provide a comprehensive view of this process. Initially, 2–3 protomers of RecA/Rad51 were involved in dynamic nucleation formation along ssDNA at multiple sites. Fast growth of RecA/Rad51 nucleation sites along ssDNA is accompanied by the stretch of ssDNA to 1.5 times its original length without ATP hydrolysis (11,12,25). Then, the presynaptic complex binds and slides along the dsDNA template to search for a homologous region until recognizing and forming stable interactions with an 8 nt-microhomology region, achieved by Watson–Crick base pairing between the invading strand and the complementary strand (26–28). The complementary strand and the displaced strand in the dsDNA template exhibit distinctive extension properties, facilitating rapid searching, recognition and strand exchange (29–31). Because mismatches occurring during homologous recognition impede strand exchange, the difference between Dmc1 and Rad51 mediated strand exchange has been examined by quantifying their binding affinities with dsDNA of different lengths of microhomology with and without mismatches (16,17,32). However, the molecular mechanism of the distinctive mismatch tolerance of these two recombinases remains elusive.

In this work, we used cryo-EM to determine the structures of hRAD51 and yeast Dmc1 (ScDmc1) with DNAs in presynaptic and postsynaptic states. Comparison of their different structures identified key residues V273 and D274 in hRAD51, corresponding to P274 and G275 for hDMC1 in the Loop2 region. We further applied single-molecule fluorescence resonance energy transfer (smFRET) assays to determine the function of mutants of hRAD51 and hDMC1 generated by swapping the two key residues mutually and to investigate their effects on strand exchange efficiency and mismatch tolerance. The structural and functional analysis led to a model that elucidates the mechanism of mismatch tolerance in detail to distinguish the property and function of the two recombinases during DNA strand exchange.

## MATERIALS AND METHODS

### Protein purification

The hRAD51 wt construct was a gift from Dr Patrick Sung (UT Health San Antonio). The hRAD51 mutants 273-PD-274, 273-VG-274, 273-PG-274, 273-VK-274 and 273-VN-274 were constructed following instructions of the point mutation kit Fast Mutagenesis System (#FM111-01, TransGen Biotech). The hRAD51 wt and mutant proteins were purified as described previously (33). Transformed *Escherichia coli* bacteria cells were grown at 37°C until the OD_600_ reached 0.6–0.8, and protein expression was induced by the addition of 0.2 mM IPTG at 37°C for 4 h. Four grams of the RecA-deficient BLR(DE3) plysS *E. coli* strain (#WR4471, Huayueyang Company) /pET21d-RAD51 dry cell pellet was lysed in 20 ml lysis buffer (100 mM Tris-OAc pH 7.5, 0.5 mM EDTA, 10% glycerol (v/v), 0.01% IGEPAL (#238539, Sigma-Aldrich), 1 pill of protease inhibitor cocktail (#04693123001, Roche), 1 mM dithiothreitol (DTT), 0.05 mg/ml lysozyme with 10 min of ultrasonic disruption. The supernatant was collected into a dialysis bag (10,000 MW cut-off) after centrifugation of the cell lysate at 38,750 × *g* and 4°C for 1.5 h.

Freshly made precipitation buffer 20 mM Tris-OAc pH 7.5, 7 mM spermidine (#124-20-9, ACMEC Biochemical) dissolved in acetate pH 7.5, 5% glycerol, 1 mM DTT was used to precipitate hRAD51 proteins overnight. The protein precipitate was collected by centrifugation at 38,975 × *g* for 20 min at 4°C. The pellet was dissolved in 10 ml T150 buffer (50 mM Tris-HCl, 150 mM KCl) by gently pipetting on ice and the soluble fraction was collected. This step was repeated in consecutive 10 ml T250, T300, T500 and T600 buffer (50 mM Tris-HCl, 250 mM KCl/300 mM KCl/500 mM KCl/600 mM KCl, respectively). Soluble fractions T150 and T250 had high levels of impurities, whereas soluble fractions T350, T500 and T600 contained purer hRAD51 proteins. The hRAD51-containing fractions were pooled together and loaded into a 20 ml MacroHap (#1572000, BioRad). The hRAD51 proteins were eluted in a KH_2_PO_4_ gradient (buffer A: 0.1 M KCl, 0.1 M KH_2_PO_4_, 10% glycerol, 1 mM DTT; buffer B: 1 M KCl, 1 M KH_2_PO_4_, 10% glycerol, 1 mM DTT). The fraction at 0.5 M KH_2_PO_4_ was collected and reloaded into 3 ml Heparin (#45-000-058, GE Healthcare) to remove the endogenous nucleotides. The fraction was further loaded to a 1-ml MonoQ (#17-5166-01, GE Healthcare), and hRAD51 was collected at ∼0.3 M KCl by gradient elution. The hRAD51 proteins with purity above 99% were flash-frozen and stored at −80°C (Supplementary Figure S1A).

ScDmc1 wt construct pNRB150 was a gift from Dr Zhi Qi (Peking University, Beijing). The plasmid containing His_6_-Dmc1 under a T7 promoter was also induced for overexpression in the RecA-deficient BLR(DE3) plysS *E. coli* strain (34). The cells were grown at 37°C until the OD_600_ reached 0.6–0.8 and protein expression was induced by adding 0.2 mM IPTG at 37°C for 4 h. Dry cells (16 g) were resuspended in 100 ml lysis buffer (25 mM Tris-HCl, pH 7.5, 10% glycerol, 500 mM KCl, 0.01% IGEPAL, 10 mM imidazole, 1 mM DTT, 1 pill of protease inhibitor cocktail) and lysed using a French press. After centrifuging at 40,000 × *g* for 1 h at 4°C, the supernatant was incubated with Talon beads (#635502, Clontech) and washed with lysis buffer containing 20, 30 and 50 mM imidazole, respectively (8). Then, ScDmc1 was eluted with elution buffer (25 mM Tris-HCl, pH 7.5, 10% glycerol, 500 mM KCl, 200 mM imidazole, 0.01% IGEPAL, 1 mM DTT, 1 pill of protease inhibitor cocktail). The eluted protein was collected and loaded into a 1-ml Heparin by applying a 10 ml gradient of 50–600 mM KCl in buffer A (50 mM Tris-HCl, pH 7.5, 100 mM KCl, 10% glycerol, 1 mM DTT, 0.01% IGEPAL (CA-630 I3021-50ML) and buffer B (25 mM Tris-HCl, pH 7.5, 1 M KCl, 10% glycerol, 1 mM DTT, 0.01% IGEPAL). The ScDmc1-containing fractions were pooled and collected at about 300 mM KCl. The eluted His_6_-ScDmc1 was concentrated to 1 mg/ml, flash-frozen and stored at −80°C (Supplementary Figure S1A).

For hDMC1 protein wt construct, the corresponding genes were synthesized by QingLan Biotech and constructed into a pET28a vector to generate a protein construct with an N-terminal His_6_-tag. The hDMC1 mutation variants 274-VG-275, 274-PD-275 and 274-VD-275 were constructed by site-directed mutagenesis as for hRAD51. Procedures of the expression and purification of hDMC1 proteins (Supplementary Figure S1A) were the same as those of ScDmc1 (8), which were described above.

### Labeled DNA preparation

DNA oligonucleotides (Supplementary Table S1) were purchased from Sangon Biotech (Shanghai, China). The Cy3- and Cy5-labeled ssDNAs were prepared via covalently conjugating the N-hydroxysuccinimido (NHS) group of fluorescent dyes to an amine group on DNA following procedures from the manufacturers. Briefly, synthesized DNA oligos were diluted to 200 μM with 100 mM NaHCO_3_ and mixed with 5 mM fluorophores (Lumiprobe), which was then incubated overnight at room temperature. Labeled DNAs were separated from excess free fluorophores through ethanol precipitation three times. The dsDNAs for homologous pairing reactions and smFRET assays were prepared by mixing the fluorescent dye labeled oligos (Supplementary Table S1) and the complementary unlabeled oligos in a molar ratio of 1.2:1 and heating the mixture to 85°C for 15 min followed by cooling to room temperature slowly.

### Homologous DNA pairing assay

The hRAD51 DNA pairing assay was assembled in buffer (25 mM Tris-HCl, pH 7.5, 25 mM KCl, 1 mM DTT, 2 mM MgCl_2_) that contained 2 mM Adenosine 5′-(β,γ-imido) triphosphate (AMP-PNP) in a final volume of 12.5 μl. The 99-mer oligo 1 (6 μM nucleotides in Supplementary Table S1) was incubated with hRAD51 wt and variants (2 μM) at 37°C for 5 min, following by the addition of 0.2 μM Hop2-Mnd1 and a 5-min incubation. The Dmc1 DNA pairing assay is similar to hRAD51 group except for 2 mM Ca^2+^ and 2 mM ATP. Then Cy5-labeled homologous dsDNA (oligo 4/oligo 5; 6 μM base pairs) was added to initiate the pairing reaction. After a 10-min incubation, the reactions were mixed with an equal volume of 1% SDS containing 1 mg/ml proteinase K (#1996204, Invitrogen). After a 5-min incubation, the reaction mixtures were resolved in 10% nondenaturing polyacrylamide gels in TBE buffer (#00006991-110451, Monad) on ice (35). The gels were photographed by fluorescent gel scanner at 699 nm, and the products were quantified by ImageJ (Supplementary Figure S1B).

### DNA strand-exchange assay

The 42-mer oligo 14 ssDNA (12 μM nucleotides in Supplementary Table S1) was incubated with hRAD51(4 μM) variants individually to form presynaptic complex in buffer (35 mM Tris-HCl, pH 7.5, 50 mM KCl, 1 mM DTT, 2 mM MgCl_2_, 2 mM AMP-PNP, 0.1% BSA) in 12.5 μl at 37°C for 10 min incubation. About 2 mM ATP and 2 mM Ca^2+^ were used for the hDMC1 variants (4 μM) to replace Mg^2+^ and AMP-PNP for hRAD51 group. For both hRAD51 and hDMC1 variants, 0.4 μM Hop2-Mnd1 was added for 10-min incubation at 37°C to increase the strand exchange efficiency. Then, exchange reaction initiated by adding 3′-Cy3 labeled mismatch-containing dsDNA (14 μM nucleotides; 8-nt paired: oligo 10/oligo 11; 6-nt paired: oligo 12/oligo 13; 6-6 nt paired: oligo 15/oligo 16) and incubated for following 15 min. The interactions were terminated by adding equal volume of 1% SDS containing 1 mg/ml proteinase K for another 15-min incubation. The samples were fractionated in 15% nondenaturing polyacrylamide gels in TBE buffer. The gels were photographed by fluorescent gel scanner at 605 nm, and the products were quantified by ImageJ (Supplementary Figure S4E–K).

### Cryo-EM samples preparation and data acquisition

The ScDmc1 presynaptic complex (ScDmc1-ssDNA) was formed by incubating 4 μM ScDmc1 wt with 150-mer ssDNA oligo 4 (12 μM nucleotides, synthesized by IDT, Supplementary Table S1) in a buffer containing 35 mM Tris-HCl, pH 7.5, 96 mM KCl, 2 mM ATP, 5 mM CaCl_2_ and 1 mM DTT at 37°C for 30 min. For postsynaptic complex (ScDmc1-dsDNA), 0.4 μM Hop2-Mnd1 was then added into the presynaptic reaction for another 10-min incubation. Homologous 150-bp dsDNA (oligo 4/oligo 5; 14.4 μM base pairs, synthesized by IDT, Supplementary Table S1) was added and incubated for 15 min at 37°C to form the postsynaptic complexes.

**Figure 1.**
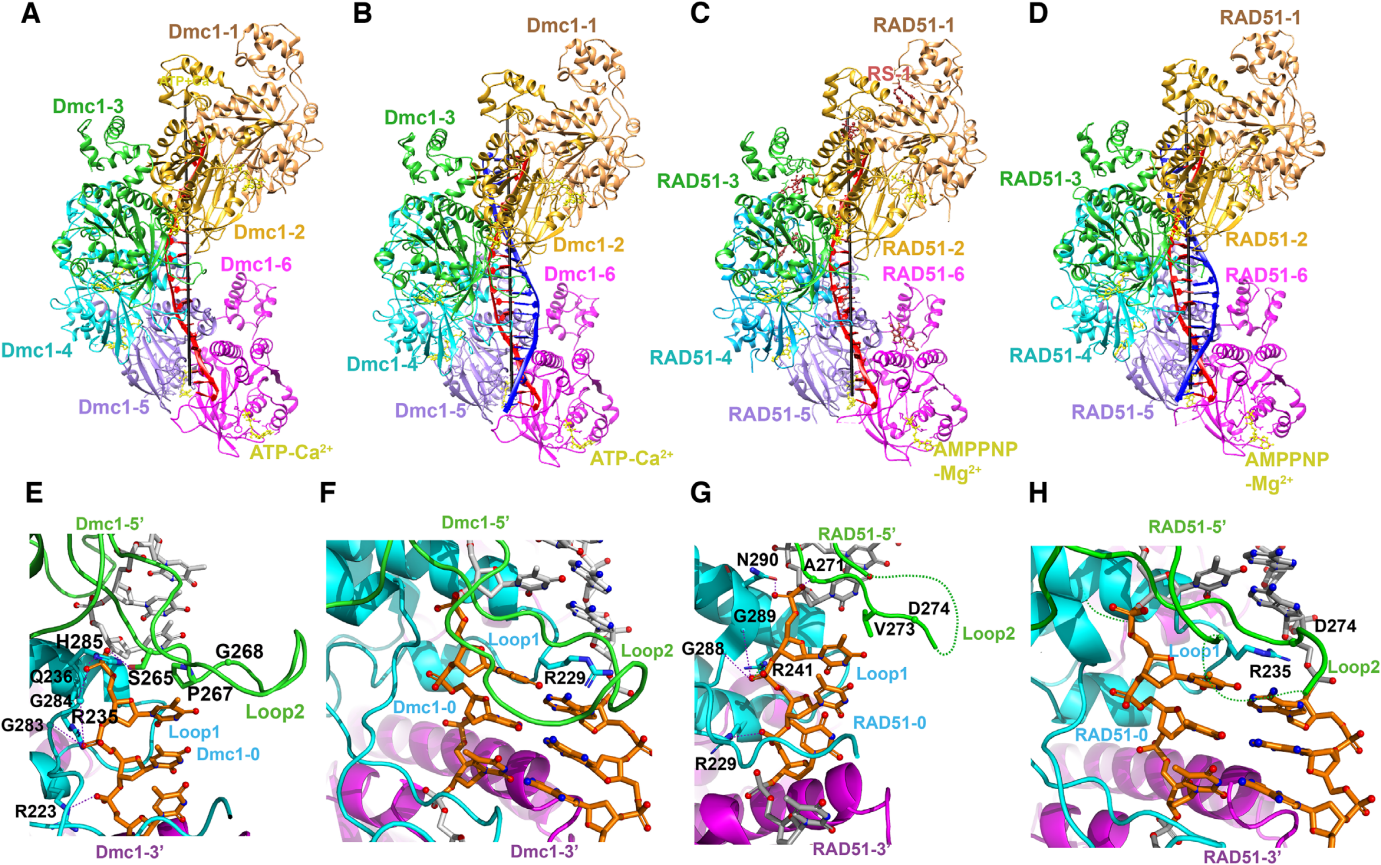
Cryo-EM structures of ScDmc1–DNAs and hRAD51–DNAs complexes. (**A**–**D**) The atomic models of ScDmc1 presynaptic complex (A) and postsynaptic complex (B), hRAD51 presynaptic complex with RS-1 (C) and postsynaptic complex without RS-1 (D) were all built based on the corresponding cryo-EM density maps. For each complex, the six protomers in one helical turn are colored orange, gold, lime green, cyan, purple and magenta. The invading strand DNA is in red while the complementary strand DNA is in blue and ATP-Ca^2+^ or AMP-PNP-Mg^2+^ is in yellow. For clarity, we named the three consecutive ScDmc1 protomers as Dmc1-5′ (green), Dmc1-0 (cyan) and Dmc1-3′ (purple) and the same for hRAD51. The chemical compound RS-1 is labeled in (C) between hRAD51-1 and hRAD51-2. (**E**) A zoomed-in view of the ScDmc1 presynaptic state with all the residues involved in possible protein–DNA interactions labeled. P267 in the Loop2 region of ScDmc1 facilitates the separation of adjacent triplets and stabilizes the triplets. (**F**) A zoomed-in view of the ScDmc1 postsynaptic state with R229 in Loop1 labeled, which interacts with the complementary strand and stabilizes the postsynaptic state. (**G** and **H**) The corresponding views of (E and F) in hRAD51 presynaptic and postsynaptic complexes, respectively. Key residues for DNA interactions are labeled. The green dash line represents for missing peptides 278-MFAA-281 in hRAD51 Loop2.

As to the hRAD51 wt presynaptic complex, 2 μM hRAD51 wt, 150-mer oligo 4 (6 μM nucleotides) and 2 μM chemical compound RS-1 that was only used for this state (for further hRAD51 stabilization) (36) (Figure 1C) were incubated in 12.5 μl assembling buffer (25 mM HEPES, pH 7.5, 25 mM KCl, 4 mM MgCl_2_ and 4 mM AMP-PNP) at 37°C for 30 min. On the other hand, the postsynaptic complex of hRAD51 wt was formed by 0.2 μM Hop2-Mnd1 and homologous dsDNA (oligo 4/oligo 5; 7.2 μM base pairs) to presynaptic complex without RS-1 to trigger strand exchange (22). The postsynaptic complex of hRAD51 273-PG-274 assembling was similar to hRAD51 wt, and RS-1 was also withdrawn from 273-PG-274 postsynaptic complex formation.

Aliquots of 4–5 μl ScDmc1 wt or hRAD51 wt reaction mixture were placed on 300 mesh Quantifoil Au R1.2 /1.3 grids coated with a graphene film (37) and flash-plunged into liquid ethane cooled down by liquid nitrogen in a Vitrobot Mark IV (Thermo Fisher Scientific) with a blotting time of 2.5 s and blotting force of −2. All frozen grids were subsequently transferred to Titan Krios microscopes (Thermo Fisher Scientific) to collect cryo-EM data. The ScDmc1 presynaptic complex (Supplementary Figure S1D and F) and the hRAD51 presynaptic complex (with RS1) (Supplementary Figure S1E and H) were collected on a Titan Krios operated at 300 keV equipped with a Cs-corrector and a Falcon II camera (Thermo Fisher Scientific) with movie mode using AutoEMation2 (developed by Jianlin Lei from Tsinghua University) at a magnification of 75,000, yielding a pixel size of 0.885 Å. The specimens were collected for their cryo-EM images with a movie exposure mode of 30 frames in 1.8 s corresponding to a total dose of 48 e^−^ Å^-^^2^ under defocus ranging from −1.0 to −3.0 μm. We collected 2,175 movie stacks for the ScDmc1 presynaptic complex and 1,159 movies for the hRAD51–RS1 presynaptic complex.

The ScDmc1 wt and hRAD51 wt postsynaptic data were collected on a Titan Krios operated at 300 keV equipped with K2 Summit direct electron-counting camera (Gatan). A total of 1,502 movies of ScDmc1 postsynaptic (Supplementary Figure S1G) complex were collected automatically with eTas software (developed by Bo Shen from Dr Xueming Li’s laboratory at Tsinghua University) at a nominal magnification of 29,000 with K2 super-resolution mode, yielding a pixel size of 1.025 Å with defocus ranging from −1 to −3.5 μm. Each movie stack was exposed for 4.8 s with an exposure time of 0.15 s per frame, resulting in a total of 32 frames per stack. The total dose was about 48 e^−^ Å^−2^ for each stack (Supplementary Figure S1D and E). For the hRAD51 postsynaptic complex (Supplementary Figure S1I), 528 movies were collected with pixel size of 1.306 Å and −1 to −3.5 μm defocus range. The total dose was about 50 e^−^ Å^-^^2^ for each stack (Supplementary Figure S1D and E) (22).

The dataset of hRAD51 273-PG-274 postsynaptic complex (Supplementary Figure S1J), comprising 2,082 movie stacks, was collected on Titan Krios G3i operated at 300 keV equipped with K3 detector and a GIF Quantum energy filter (slit width 20 eV) automatically using AutoEMation2 (developed by Jianlin Lei from Tsinghua University). The pixel size is 1.0825 Å with defocus ranging from −1.5 to −2.5 μm. The total dose was about 40 e^−^ Å^−^^2^ for each stack.

### Cryo-EM image processing

Raw data of ScDmc1 presynaptic complex acquired from the Falcon II camera were converted to MRC format by a locally written program, Raw2MRC. The MRC stacks were first aligned and summed with MotionCor2 (38) and binned two-fold with dose weighting applied. The CTF parameters were determined with Gctf (39). After CTF determination and evaluation, the ScDmc1 presynaptic complex dataset with 2,154 micrographs was selected for further processing. A total of 290,031 particles were eventually segmented automatically using Relion 2.1 (40,41). After 2D classification, 225,795 particles were subjected to the 3D auto refine procedure and the final reconstruction was obtained at resolution of 3.2 Å (Supplementary Figure S2A). For the hRAD51–RS1 presynaptic complex, 1,115 micrographs were selected after CTF determination and evaluation, from which 321,427 particles were picked automatically with Relion 2.1. Eventually, the final reconstruction was obtained at a resolution of about 2.97 Å (Supplementary Figure S2B) by following a processing strategy described before (22).

The output MRC stacks of ScDmc1 or hRAD51 postsynaptic complex (22) collected by eTas software were motion-corrected with MotionCor2 and binned two-fold with dose weighting applied. The non-dose-weighted images were used for CTF estimation by Gctf while the dose-weighted ones were used for particle picking and reconstruction. In total, 112,493 ScDmc1 postsynaptic complex particles were picked automatically in Relion 3.0 (42). After 2D classification, 107,513 particles showing clear features were retained for 3D classification and reconstruction. A total of 71,192 particles were selected from the local angular search and subjected to further CTF and beam tilt refinement (43), after which another round of 3D auto refine was performed, resulting in a 3D reconstruction with overall resolution of 3.4 Å (Supplementary Figure S2C). The hRAD51 postsynaptic complex was reconstructed in a similar way to achieve better reconstruction with a resolution of 3.98 Å (Supplementary Figure S2D) than the previously published one (EMD-9567).

Raw data of hRAD51 273-PG-274 postsynaptic complex acquired from the K3 detector were converted to MRC format by a locally written program ‘TsinghuaTitan.py’. The MRC stacks were first summed with MotionCor2 and binned two-fold with dose weighting (Supplementary Table S2). A total of 960,393 particles were segmented automatically using Relion 3.0. After 2D classification, 757,143 particles were subjected to the 3D classification and 139,616 particles eventually entered into 3D auto-refine, resulting a final reconstruction at 3.0 Å (Supplementary Figures S2E, S3N–O).

### Model building and structure refinement

The initial atomic model of the ScDmc1 presynaptic complex was generated from the cryo-EM structure of hRAD51 (22) (PDB accession number: 5H1B) by CHAINSAW (44) and was rigid-body docked into the electron density map in UCSF-Chimera (45). The Loop2 region involved in DNA binding is highly flexible and therefore the amino acids in Loop2 were built manually based on the density map in COOT (46). The atomic models of the longer assemblies were generated by copying the protomer several times in UCSF-Chimera following their corresponding helical symmetries. After manual adjustment of every residue in COOT, the structures were further refined in real space in PHENIX with secondary structures and geometric restraints (47) to obtain the models of the presynaptic and postsynaptic complexes.

For the hRAD51–RS1 presynaptic complex, the published hRAD51–ssDNA structure (22) (PDBID: 5H1B) was first docked into the EM density map and then through the whole map. In COOT, certain positions of the side chains were manually adjusted. With some real-space refinement, the final atomic model of the hRAD51 presynaptic complex was obtained. Most of the structure of the hRAD51 mutant 273-PG-274 postsynaptic complex was similar to hRAD51 wt. The density of the Loop2 region of 273-PG-274 was more rigid than wt, although the Loop2 region side chains of the former were still not solid enough to build a model, indicating the highly flexible nature of Loop2 region in hRAD51 (Supplementary Figure S3N–O).

### Preparation of PEG-passivated slides

PEG-passivated slides were prepared according to a previous procedure with minor modifications (48). In brief, slides and coverslips (Thermo) were sonicated at 40°C sequentially in the order of ethanol (10 min), 0.2 M KOH (20 min) and ethanol (10 min). Cleaned slides and coverslips were treated with amino-silane reagents (1 ml of 3-aminopropyltriethoxysilane, 5 ml of acetic acid and 94 ml of methanol) at room temperature overnight and then incubated with polyethylene glycol (PEG from Laysan Bio, Inc., containing 20% w/w mPEG-Succinimidyl Valerate, MW 2,000 and 1% w/w Biotin-PEG-SC, MW 2,000) in 0.1 M sodium bicarbonate (pH 8.3) for 3 h. Slides and coverslips were dried using clean N_2_, put in 50 ml falcon tubes, vacuum-sealed in food saver bags and stored at −20°C.

### Acquisition of smFRET data

PEG-passivated slides were incubated with 0.05 mg/ml streptavidin for 2 min. The 5′-Cy5-labeled invading DNA (oligo 6/oligo 7; 50 pM strand concentration) was prepared in buffer A (50 mM Tris-HCl, pH 7.5, 96 mM KCl, 2 mM MgCl_2_, 1 mM AMP-PNP and 1 mM DTT) and then fixed on the streptavidin coated surface via the specific non-covalent interactions between streptavidin–biotin. The hRAD51 (hDMC1) variants were diluted to 500 nM in buffer A, injected into immobilized invading DNA and incubated for 5 min, and the unbound protein was washed out with buffer A. Then 50 nM Hop2-Mnd1 in buffer A was added to immoblized RAD51-ssDNA before flowing dsDNA to increase strand exchange efficiency. Then, the different duplex CD strands (fully paired oligo 8/oligo 9; 1 nM strand concentration), 8 nt-paired (oligo 10/oligo 11; 1 nM strand concentration), 6 nt-paried (oligo 12/oligo 13; 1 nM strand concentration) and 6–6nt paired (oligo 15/oligo 16; 1 nM strand concentration) dsDNAs were injected into channels to initiate the strand exchange process while recording fluorescence signals. All smFRET experiments were performed at 30°C in buffer A with an oxygen scavenging system, containing 3 mg/ml glucose, 100 μg/ml glucose oxidase, 40 μg/ml catalase (Roche), 1 mM cyclooctatetraene, 1 mM 4-nitrobenzylalcohol and 1.5 mM 6-hydroxy-2,5,7,8-tetramethyl-chromane-2-carboxylic acid.

Single-molecule fluorescence and FRET measurements were performed on a home-built objective-type TIRF microscope, whose configuration details were described previously (48). All smFRET movies were collected using Cell Vision software (Beijing Coolight Technology). The apparent FRET efficiency (*E*_app_) is defined as *E*_app_ = *I*_A_/(*I*_A_ +*I*_D_), in which *I*_A_ stands for intensity of acceptor Cy5 and *I*_D_ stands for intensity of donor Cy3.

### Quantification of strand exchange efficiency and binding rate constant

After strand exchange, the number of single-molecule fluorescence spots per imaging field under 532 and 640 nm laser excitation was separately quantified. The number of Cy5 spots under 640 nm laser excitation (*N_Cy5_*) defined the number of immobilized strand I, whereas the number of FRET spots under 532 nm laser excitation (*N*_FRET_) represented the number of strand I bound with stand C caused by strand exchange. Thus, strand exchange efficiency was calculated as *N*_FRET_/*N*_Cy5_ ratio. A three-stranded DNA complex was formed by mixing invading strand (oligo 6/oligo 7) and fully complementary strand C (oligo 8) at a ratio of A(Cy5):I(biotin):C(Cy3) = 1.2:1:1.2 (strand concentration) to mimic an ideal exchanged postsynaptic sample. The value of *N*_FRE_*_T_*/*N*_Cy5_ of this ideal three-stranded DNA complex was used as the standard value to normalize other measured *N*_FRET_/*N*_Cy5_ ratios. According to our smFRET assay, strand exchange efficiency of hRAD51 wt is 25 ± 1% which is similar to previously reported values (22,49).

While capturing strand exchange in real time, the appearance time (*t*_app_) of homologous dsDNA onto protein-coated-ssDNA during the strand exchange process was extracted from accumulative counts via single exponential decay fitting as shown in Figure 3I and J. The binding rate constant (*k*_app_) of dsDNA (Table 1) was calculated via *k*_app_ = 1/(*t*_app_•[CD]), in which [CD] is the concentration of dsDNA (oligo 8/oligo 9, 1 nM strand concentration) flowing into the channels for homologous strand exchange.

**Table 1.** Exchange rates of hRAD51/hDMC1 with fully paired DNA

***k*_app_ / μM^−1˙^s^−1^**	**273-VD-274**	**273-PD-274**	**273-VG-274**	**273-PG-274**	**273-VN-274**	**273-VK-274**
**hRAD51**	25 ± 1	28 ± 3	117 ± 12	164 ± 20	124 ± 9	106 ± 20
	**274-VD-275**	**274-PD-275**	**274-VG-275**	**274-PG-275**	**–**	**–**
**hDMC1**	27 ± 2	24 ± 3	108 ± 5	110 ± 10	–	–

### Molecular dynamic simulation

The initial coordinates of the DNA models with wt of hRAD51 and hDMC1 were built based on the models obtained from the cryo-EM structures of hRAD51 and ScDmc1 DNA complexes. The missing residues of Loop2 in hRAD51 were constructed using Modeller9.20 (50), and three different initial conformations of Loop2 in hRad51 were selected for molecular dynamic simulations. Three DNA models were used in the simulations. One contains the homologous 9 bp-paired dsDNA (oligo 17/oligo 18 and Supplementary Table S1), 8 bp-paired dsDNA (the second chain sequence was changed to 5′-AAAAGAAAA-3′ (oligo 17/ oligo 19 and Supplementary Table S1) and ssDNA. Based on the simulations of wt hRAD51 and hDMC1, the mutations 273-PD-274, 273-VG-274, 273-PG-274, 273-VK-274, and 273-VN-274 of hRad51 and 274-VG-275, 274-PD-275, and 274-VD-275 of hDMC1 with DNA models were built to investigate the function of these residues.

Atomistic molecular dynamic simulations of initial models were carried out in the AMBER16 program using AMBER14SB force field for protein (51) and Parmbsc1 force field for DNA (52), and the parameters were obtained from the parameters reported previously (53) for ATP. Each system was neutralized with a number of magnesium ions and then immersed in a solvent box filled with TIP3P water molecules (54) to warrant a distance of at least 20 Å between the surface of each protein–DNA models and the water box edge. Energy minimization was performed by imposing a strong restraint on each system and was followed by minimizing the whole system for a few thousand steps. NVT (constant number of atoms, volume and temperature) simulations were carried out by heating the whole system slowly from 100 to 300 K, and the Berendsen thermostat (55) was used to maintain the temperature of the whole system. Subsequently, 1 ns NVT dynamics was performed and was followed by a NPT (constant number of atoms, pressure and temperature) production run. During the NPT production run, all bonds associated with hydrogen atoms were constrained by employing the SHAKE algorithm (56) such that the integration time step of 4 fs could be used. A cutoff value of 12 Å was set for nonbonded interactions, and the Particle Mesh Ewald method (57) was employed for treating electrostatic interactions. For each system, five independent molecular dynamic simulations were carried out using different velocities that were randomly generated at the beginning of the simulations and run for 1 μs. The analysis of each molecular dynamic trajectory was performed with the cpptraj module in Amber 16 (58).

### MM-GBSA calculation

To understand the interaction between the DNA molecules with hRAD51 and hDMC1, the binding free energies were calculated using the MM-GBSA method. For each complex, 500 snapshots were extracted from the last 50 ns along the molecular dynamic trajectory at an interval of 100 ps. The MM-GBSA method (59) was performed to compute the binding free energies of the substrates with each mutant. The binding free energy ($\Delta G$) can be represented as:

$$\begin{equation*}\Delta G\; = \;\Delta {E_{MM}} + \Delta {G_{sol}}\end{equation*}$$

where $\Delta {E_{MM}}$ is the difference of molecular mechanic energy between the complex and each binding partner in the gas phase, $\Delta {G_{sol}}\;$is the solvation free energy contribution to binding and $T\Delta S$ is the contribution of entropy changes to the binding free energy. $\Delta {E_{MM}}$ is further divided into two parts:

$$\begin{equation*}\Delta \;{E_{MM}} = \;\Delta {E_{ele}} + \Delta {E_{vdW}}\end{equation*}$$

where $\Delta {E_{ele}}$ and $\Delta {E_{vdW}}$ are described as the electrostatic interaction and van der Waals energy in the gas phase, respectively. The solvation free energy is expressed as:

$$\begin{equation*}\Delta \;{G_{sol}} = \;\Delta {G_{gb}} + \Delta {G_{np}}\end{equation*}$$

where $\Delta {G_{gb}}$ and $\Delta {G_{np}}$ are the polar and non-polar contributions to the solvation free energy, respectively.

## RESULTS

### Cryo-EM structures of presynaptic and postsynaptic complexes of two recombinases

Using procedures described previously (22), we obtained cryo-EM structures of the presynaptic and postsynaptic complexes of ScDmc1 wt at the resolutions of 3.2 and 3.4 Å, respectively, and those of hRAD51 wt at 3.0 and 4.0 Å, respectively (Supplementary Figures S1 and S2); these resolutions were higher than for previously reported structures (22). All four helical assemblies share almost the same right-handed helical symmetry with about 6.3 protomers per turn and a helical pitch of 100 Å (Figure 1A–D). In our models, ssDNA and dsDNA substrates are located in the central axis of the helical structures and stretched to form continuous triplets with DNA bases exposed for homologous pairing and search.

Comparing ScDmc1 assembly structures with those of hRAD51 demonstrates the high similarity of the interacting interfaces between neighboring protomers (Supplementary Figure S3). The first interaction is formed with the ATP binding pocket buried in the neighboring protomer’s C-terminal portion. ScDmc1, which is highly conserved with RecA, ScRad51 and hRAD51, has similar Walker A and B motifs for ATP hydrolysis and the surrounding hydrogen bonds network for coupling of ATP binding and DNA interaction (Supplementary Figure S3A). The second interaction is the beta strand formed by a linker region of one protomer pairing with the beta sheet in the ATPase core domain of the adjacent protomer (Supplementary Figure S3B), which is also conserved in hRAD51 assemblies (20,22,60,61).

The structures also demonstrate a generally similar mode of DNA interaction of hRAD51 and ScDmc1 in the presynaptic and postsynaptic complexes, both involving the Loop1 and Loop2 motifs of the protein protomers (Figure 1E–H and Supplementary Video S1). In the ScDmc1 presyaptic complex (Figure 1A) and hRAD51 presynaptic complex with RS-1 (Figure 1C) which is a compound could enhance the DNA exchange activity (36,62), one DNA nucleotide-triplet in a B-form conformation interacts with two consecutive protomers. More specifically, the triplet is sandwiched by Loop2 of ScDmc1-5′ and ScDmc1-0 in the helical axial direction, as well as by Loop1 and Loop2 of ScDmc1-0 in the vertical direction. In ScDmc1 (Supplementary Figure S3E–G), the first phosphate within the triplet is bound by S265 of Dmc1-5′, Q236 and H285 of Dmc1-0, the second phosphate interacts with G283, G284 and R235 of Dmc1-0, and the third phosphate interacts with R223 of Dmc1-0 (Figure 1E). All of these residues are highly conserved in hRAD51 (22). In the postsynaptic complexes of both hRAD51 and ScDmc1, Watson–Crick base pairs are formed between the complementary strand and invading strand in triplet clusters. R229 in Loop1 of ScDmc1 stabilizes the complementary strand (Figure 1E–G and Supplementary Figure S3H–I) while its equivalent R235 of hRAD51 (Figure 1H) plays the same role (22).

### Distinctive Loop2 regions between hRAD51 and ScDmc1

**Figure 2.**
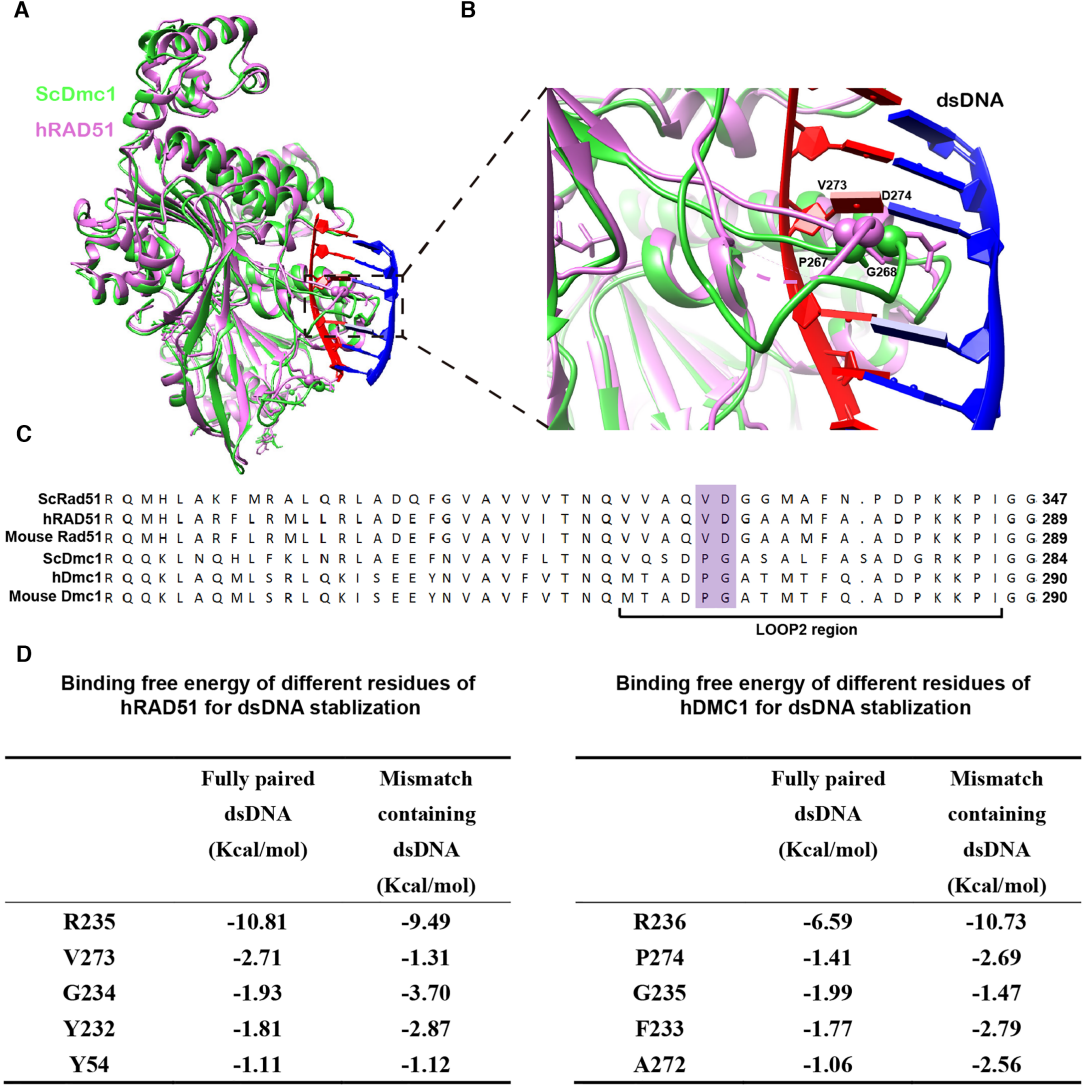
The structural difference of hRAD51 and ScDmc1 in the Loop2 region. (**A**) The postsynaptic complex of hRAD51 (pink) and ScDmc1 (green) are superimposed in two consecutive protomers. (**B**) The Loop2 of hRAD51 and ScDmc1 with the DNA substrate are shown in an enlarged view of (A). The α-carbon atoms are represented by spheres for V273 and D274 of hRAD51, and P267 and G268 of ScDmc1, in the corresponding Loop2 regions. The pink dash line represents for missing peptides 278-MFAA-281 in hRAD51 Loop2. (**C**) Sequence alignment of representative eukaryotic Rad51 and Dmc1. The residues of VD in eukaryotic Rad51 and the corresponding PG of Dmc1 in the Loop2 region are marked in purple. (**D**) The change of binding free energies of several key residues in the Loop2 and Loop1 regions in hRAD51 wt and hDMC1 wt, respectively. The top five residues with the strongest interaction are listed for each protein.

The major structural difference between hRAD51 and ScDmc1 lies in Loop2 of the DNA-bound complexes (Figure 2). We found that the EM density corresponding to Loop2 of hRAD51 complexes is less resolved than that of ScDmc1 (Supplementary Figure S3C–D), despite the higher resolution of the hRAD51 presynaptic complex among four 3D reconstructions. The EM densities of ScDmc1 reconstructions are sufficient to build atomic models of the intact Loop2, but several residues in the Loop2 of hRAD51 cannot be accounted for in the corresponding reconstructions (Figure 2A and B), indicating a more flexible nature of Loop2 in hRAD51. We noticed that P267 of Loop2 in ScDmc1 inserts into two adjacent nucleotide triplets and stabilizes the stretched DNA strand, playing a similar role as V273 in hRAD51 (Supplementary Figure S3C and D). But proline has a more rigid main chain conformation than valine.

The observation that the hRAD51 and ScDmc1 structures are quite similar in most parts of the DNA-bound complexes but display dramatic differences in the Loop2 region (22) is in parallel to the sequence variation of Loop2 between Rad51 and Dmc1 (Figure 2A–C and Supplementary Figure S3E–I). We thus wondered whether Loop2 is responsible for the distinctive mismatch tolerance between different recombinases. The highly conserved sequence between hDMC1 and ScDmc1 allowed us to build atomic models of hDMC1 via molecular dynamic (MD) simulation based on the structure of ScDmc1 (Figure 2C), which was verified by comparison to hDMC1 structures determined by cryo-EM very recently (24) (Supplementary Figure S3J and K). To understand the role of Loop2 in DNA strand binding, MD simulation was also performed for the presynaptic and postsynaptic complexes of hRAD51 and hDMC1. We calculated the energy contribution of different residues in two loops to stabilize fully paired and mismatch-containing dsDNA substrates (Figure 2D; Supplementary Tables S3 and S4), which were estimated by using MM-GBSA calculations (details in the Materials and Methods). The results suggested that V273 and P274 of hRAD51 and hDMC1, respectively, are crucial for dsDNA binding in the Loop2 region (Figure 2D). Furthermore, we found that the Rad51-specific D274 interacts with R235 through an electrostatic interaction and stabilizes the latter's conformation (Supplementary Video S2). R235 of hRAD51 and its counterpart R236 of hDMC1 in Loop1 both interact with two bases of the invading strand through a π–π interaction to stabilize DNA substrates (Supplementary Video S2). The results of the MD simulations show that the V273 and D274 in Loop2 of hRAD51, corresponding to P274 and G275 of hDMC1, play a crucial role in the DNA binding. Together, V273 and D274 in Loop2 of hRAD51, corresponding to P274 and G275 of hDMC1, are likely to play key roles during strand exchange.

### Two residues in the Loop2 region regulate the efficiency, rate and mismatch tolerances of strand exchange

**Figure 3.**
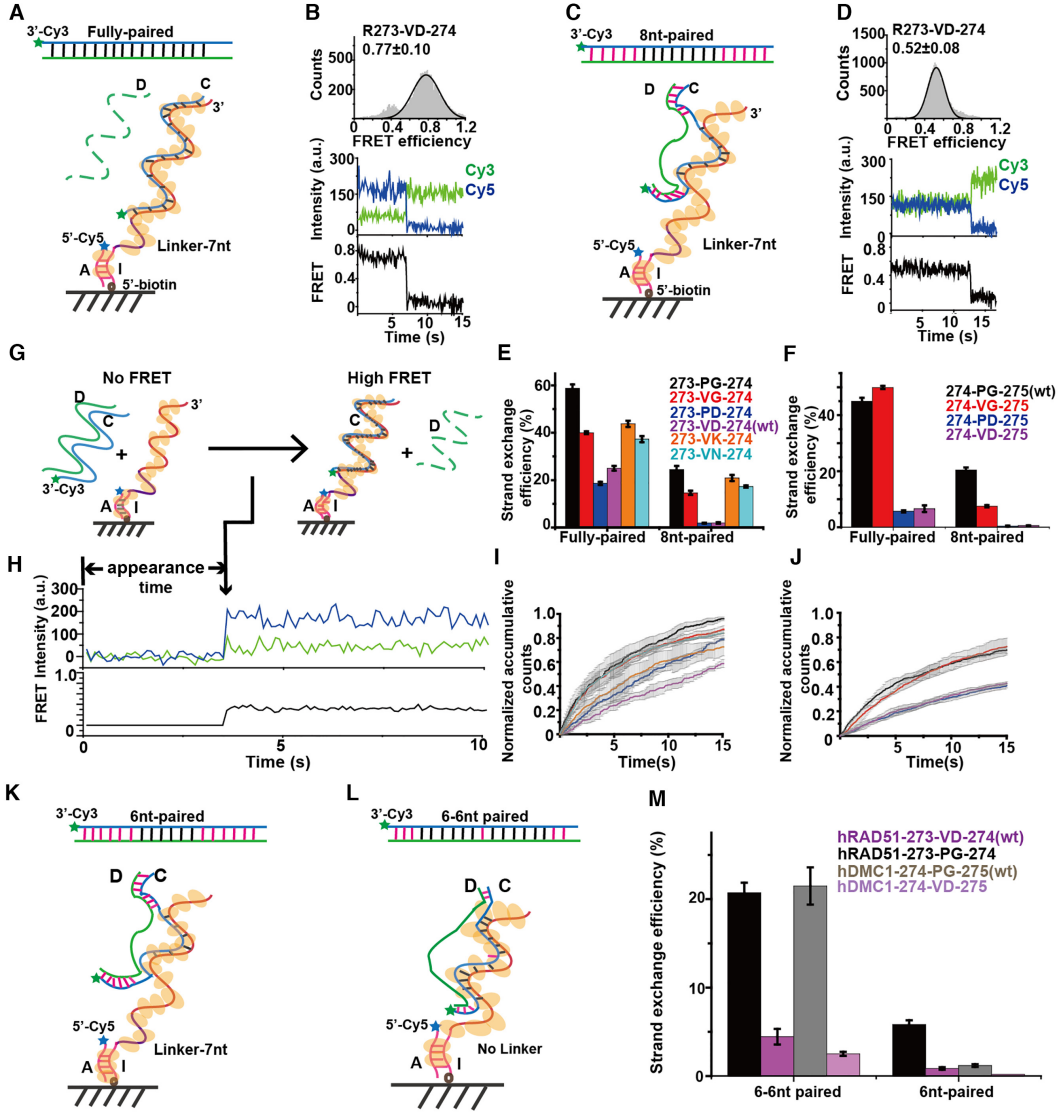
smFRET assays to quantify the efficiency and rate of strand exchange for hRAD51 and hDMC1 variants. (**A**) Cartoon illustration of strand exchange with fully paired dsDNA. (**B**) Distribution of smFRET and a corresponding single-molecule fluorescence trajectory after strand exchange mediated by hRAD51 wt with fully-paired dsDNA. R represents hRAD51. (**C**) Cartoon illustration of strand exchange with 8nt-paired dsDNA. (**D**) Distribution of smFRET and a corresponding single-molecule fluorescence trajectory after strand exchange mediated by hRAD51 wt with 8nt-paired dsDNA. (**E** and **F**) The strand exchange efficiencies mediated by hRAD51 (E) and hDMC1 variants (F). 273-VD-274 is hRAD51 wt and 274-PG-275 is hDMC1 wt. The error bars denote the SEM of six repeats of experiment. (**G** and **H**) Cartoons illustration of the smFRET assay and a corresponding single-molecule trajectory to capture strand exchange rate of fully paired dsDNA. Cy3-labeled dsDNA was injected into flow channels containing Cy5-labeled immobilized presynaptic complex while fluorescence signals of Cy3 and Cy5 were recorded. Strand exchange leads to appearance of fluorescence and FRET signals indicated by the arrow. (**I** and **J**) Time-dependent normalized accumulative counts of appeared FRET spots in the presence of fully paired dsDNA with hRAD51 (I) and hDMC1 (J) variants. Color codes are the same as in (E) and (F). The error bars denote the SEM of more than three repeats of experiment. (**K** and **L**) Cartoon illustration of strand exchange with 6nt-paired dsDNA, and an individual mismatch-containing ‘6–6nt paired’ dsDNA. (**M**) Strand exchange efficiencies of different dsDNAs of (K and L) mediated by hRAD51 wt, and 273-PG-274; and hDMC1 wt, and 274-VD-275. The error bars denote the SEM of six repeats of experiment.

Based on results of MD simulation, we sought to understand the functions of residues V273 and D274 in Loop2 of hRAD51 and corresponding P274 and G275 of hDMC1 using smFRET assays (Figure 3). We generated six constructs via mutual mutation between hRAD51 and hDMC1, including hRAD51 273-PD-274, 273-VG-274 and 273-PG-274; and hDMC1 274-VG-275, 274-PD-275 and 274-VD-275 (Table 1 and Supplementary Figure S1). The capability of strand exchange was examined by a homologous DNA pairing assay (Supplementary Figure S1B), confirming that hRAD51 and hDMC1 mutants were able to facilitate homologous strand exchange as in the wt. We then performed smFRET assays to quantify the strand exchange efficiencies of different hRAD51 and hDMC1 variants more accurately (27). The invading DNA strand (oligo 7 (5′-biotin-labeled strand I)/oligo6 (Cy5-labeled strand A); 25pM strand concentration) was immobilized on the surface of a coverslip via biotin–streptavidin conjugation, whose surface density was usually ∼1,500 molecules per imaging field, and incubated with 500 nM recombinase variants to form presynaptic complexes. Subsequently, the 18-bp Cy3-labeled dsDNA (duplex CD in Figure 3A, which was annealed by different pairs of complementary strand and displaced strand described in Materials and Methods) was added to trigger strand exchange (Figure 3A and C). When the duplex CD was completely homologous (oligo 8/oligo 9; 1 nM strand concentration) with the invading strand I (Figure 3A), we captured the appearance of a high FRET state (FRET efficiency ∼0.7, Figure 3B and Supplementary Figure S4A). Moreover, inspired by the theory of ‘8 nt-microhomology’ stabilization, duplex CD containing eight continuous homologous bases toward strand I in the middle and five non-complementary bases on each end (8nt-paired dsDNA in Figure 3C) was designed as a mismatch-containing sample. In contrast to the completely homologous sample, this mismatch-containing sample showed a decreased FRET efficiency to ∼0.5, indicating further apart of the two labeling sites (Figure 3D and Supplementary Figure S4B).

To compare the ability of hRAD51 and hDMC1 variants to mediate strand exchange with different dsDNAs, we quantified the strand exchange efficiencies via smFRET assays. Briefly, the number of Cy5 spots under 640 nm laser excitation (*N_Cy5_*) defines the total number of immobilized strand I, and the number of FRET spots under 532 nm laser excitation (*N*_FRET_) represents the number of strand C bound to strand I after the strand exchange reaction. Thus, the strand exchange efficiency can be quantified via the *N*_FRET_/*N*_Cy5_ ratio. For the exchange of fully paired dsDNA, hRAD51 mutants 273-VG-274 and 273-PG-274 exhibited higher exchange efficiencies of 40 ± 1% and 59 ± 2%, respectively, in comparison with the hRAD51 wt 273-VD-274 and mutant 273-PD-274 showing efficiencies of 25 ± 1% and 19 ± 1%, respectively (Figure 3E and Supplementary Figure S4E). Moreover, the exchange efficiencies of fully paired dsDNA were noticeably higher than that of 8nt-paired dsDNA for all constructs in hRAD51 variants. The exchange efficiencies of 273-VG-274 and 273-PG-274 with 8nt-paired dsDNA decreased by 2.7- and 2.4-fold (15 ± 1% and 24 ± 2%), respectively; whereas the exchange efficiencies of wt 273-VD-274 and 273-PD-274 with 8nt-paired dsDNA decreased by 13.2- and 10.6-fold (1.9 ± 0.3% and 1.8 ± 0.2%), respectively. These phenomena suggested that the hRAD51 mutants 273-VG-274 and 273-PG-274 were more tolerant to mismatches.

A similar trend of strand exchange efficiencies was observed among hDMC1 variants (Figure 3F and Supplementary Figure S4H). The strand exchange efficiency of hDMC1 mutant 274-VG-275 decreased 6.7-fold from 50 ± 7% with fully paired dsDNA to 7.5 ± 0.4% with 8nt-paired dsDNA, and hDMC1 wt 274-PG-274 decreased from 45 ± 1% to 20 ± 1% (2.3-fold), supporting their moderate sensitivity toward a single mismatched base pair. In contrast, the exchange efficiencies of hDMC1 274-VD-275 and 274-PD-275 with fully paired dsDNA were 6.6 ± 1.2% and 5.6 ± 0.4%, respectively. The values further decreased by 13.2- and 14-fold to 0.5 ± 0.1% and 0.4 ± 0.2%, respectively, with 8nt-paired dsDNA. The behaviors of hDMC1 mutants 274-VD-275 and 274-PD-275 are similar to hRAD51 wt 273-VD-274 and mutant 273-PD-274, i.e. less capable of triggering strand exchange and notably sensitive to mismatches. We also used the smFRET assays to quantify the exchange rates by measuring the appearance rate of stable FRET signals between strands I and C after injecting the fully paired dsDNAs to the immobilized presynaptic complexes (Figure 3G–J). Our results therefore indicated a strong correlation between the strand exchange rate and the exchange efficiency (Table 1).

To examine and quantify mismatch tolerance of hRAD51 and hDMC1 variants, a single-base mismatch was introduced in the center of a 13 nt-paired dsDNA to generate 6–6nt paired dsDNA (Figure 3K; Supplementary Figure S4C, F and I). In addition, 6nt-paired dsDNA which does not meet ‘8 nt- microhomology’ was also examined (Figure 3M; Supplementary Figure S4D, G and J). Consistent with previous data using 8nt-paired dsDNA containing mismatches in both ends, hDMC1 wt displayed higher mismatch tolerance to the mismatch containing dsDNA (6–6nt paired dsDNA) than hRAD51 wt. In addition, the constructs with PG were more likely to overcome the single-base mismatch to form stable complexes than the VD constructs (Figure 3L). Therefore, residues VD and PG strongly affect the strand exchange efficiency and mismatch tolerance of both hRAD51 and hDMC1.

### The negatively charged asparate in Loop2 of Rad51 as the key residue of strand exchange accuracy

As described above, the hRAD51 and hDMC1 variants of VG and PG both displayed higher mismatch tolerance than the variants of VD and PD, indicating that the conversion of G to D plays critical roles to regulate the strand exchange accuracy. To further understand the importance of D274 of hRAD51, we mutated D274 to lysine with an opposite electric charge (273-VK-274) or to asparagine without electric charge (273-VN-273) but both with similar side chain dimensions of the aspartate. The smFRET assay measurements showed similarly high mismatch tolerances of hRAD51 273-VK-274 (44 ± 1% with fully paired dsDNA and 21 ± 1% with 8 nt-paired dsDNA, 2.1-fold decrease) and hRAD51 273-VN-274 (38 ± 1% for fully paired dsDNA and 17.3 ± 0.5% for 8 nt-paired dsDNA, 2.2-fold decrease) as those of hRAD51 mutant 273-VG-274 and 273-PG-274, whose tolerances are significantly higher than that of hRAD51 wt 273-VD-274 (Figure 3E and Table 1). These results further emphasized that D274 in Loop2 plays a key role to restrict the exchange efficiency and to proofread exchange accuracy in hRAD51 wt, whereas other substitutes all lost the proofreading capability.

**Figure 4.**
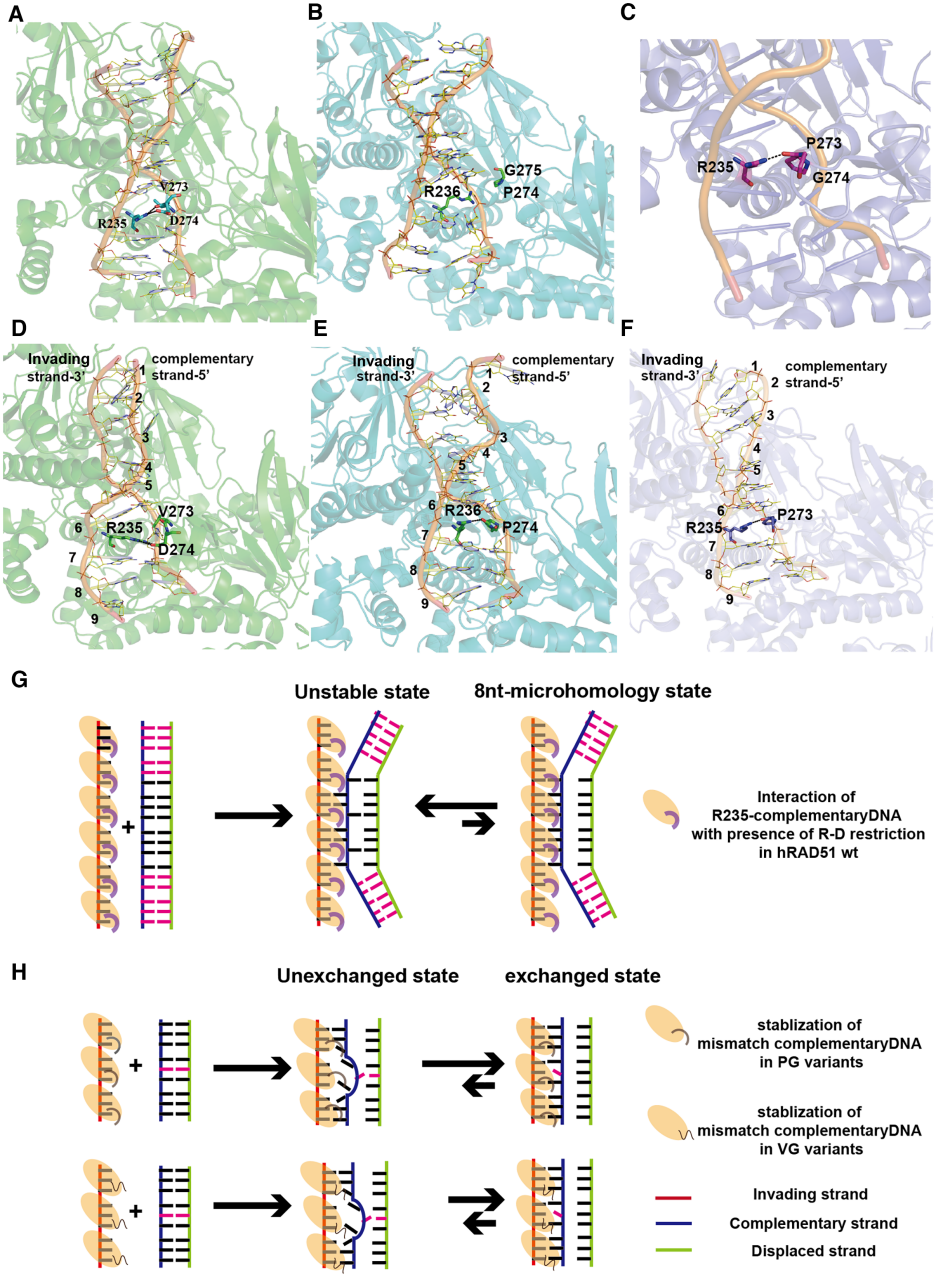
Models of the proofreading mechanism during DNA strand exchange regulated by two key residues in the Loop2. (**A**) A snapshot of the molecular dynamic simulation to show the electrostatic interaction between D274-R235 for fully paired dsDNA binding of hRAD51 wt. (**B**) A snapshot of the molecular dynamic simulation to show the disappearance of D-R electrostatic interaction for fully paired dsDNA binding of hDMC1 wt. (**C**) A snapshot of the molecular dynamic simulation to show the weak interaction between R235 and P273 for fully paired dsDNA binding of hRAD51 273-PG-274 variant. (**D**) A snapshot of the molecular dynamic simulation to show the strong restriction formed by rigid interaction of D274-R235 to prevent mismatch-containing dsDNA from binding to hRAD51 wt. (**E**) A snapshot of the molecular dynamic simulation to show the flexible interaction between R236 and P274 to support the mismatch-containing dsDNA binding of hDMC1 wt. (**F**) A snapshot of the molecular dynamic simulation to show the relatively unstable interaction between R235 and V273 to weakly support the mismatch-containing dsDNA binding of hRAD51 273-PG-274. (**G**) The cartoon model demonstrating the proofreading mechanism during the strand exchange process. The interaction between D274 and R235 (the bold curve attached to hRAD51 protomer with oval shape) could be a rigid obstruction of the third base flipping for base pairing. (**H**) The cartoon model of mechanism of conducting the mismatch-containing dsDNA exchange in PG and VG variants. Magenta dashes represent the mismatch base of the complementary strand towards the invading strand, and black dashes represent the match base pairs of the complementary and invading strand. In the absence of the D-R interaction, the PG mutant (the regular curve attached to the oval hRAD51) permits faster strand exchange leading to higher efficiency than the VG mutant (the regular wave line attached to the oval hRAD51 ).

The above results are in line with the structural analysis and MD simulation that revealed a strong electrostatic interaction of D274 in Loop2 with R235 in Loop1 (22), but D274 barely binds to DNA (Supplementary Table S3, lower contribution from D274 to stabilize DNAs for all constructs). This also indicates that the effect of D274 to mismatch tolerance is R235-dependent. Indeed, a π–π interaction of R235 to neighboring triplets is coupled with the R-D interaction in hRAD51 wt (Figure 4A). When a mismatch-containing dsDNA is recruited, the flipping-out and disordered complementary strand could easily escape from that π–π stabilization and coupled DNA binding capability of R235 disappeared because of no flexibility of R-D interaction in hRAD51 wt, resulting in conformational change of R to mismatch containing DNA triplets (Figure 4D) and low mismatch tolerance. This is also supported by the binding free energies of different residues to DNAs (Supplementary Table S3). The binding free energy of R235 clearly is higher with fully paired DNA (−10.82 kcal/mol) than with mismatched DNA (−9.46 kcal/mol) in hRAD51 wt. The same is true in hRAD51 273-PD-274 (−10.90 kcal/mol of fully paired DNA to −10.04 kcal/mol of mismatched DNA). Therefore, this R-D interaction indicates a potential barrier for stabilization of the mismatch-containing complementary strand (Supplementary Video S2). On the other hand, when the variants do not contain this negatively charged aspartate in the Loop2, the binding free energy contribution of R235 barely changed from fully paired DNA to mismatched DNA in both hRAD51 273-PG-274 (−10.03 to −10.09 kcal/mol) (Figure 4C and F) and 273-VG-274 (−8.74 to −8.28 kcal/mol) (Supplementary Figure S5B), indicating a minimal perturbation of the R235 interaction with mismatched DNA in the absence of the rigid R-D electrostatic pairing. Thus, PG and VG displayed significantly higher mismatch tolerance during strand exchange.

The similar trend could also be found in hDMC1 variants. R236 in hDMC1 wt was also observed to provide stabilization for homologous complementary strand, but this was not restrained by G275 or P274 (Figure 4B and Supplementary Table S4). However, compared with the binding free energy of the fully paired DNA, that of the mismatched DNA by R236 decreased (−6.59 to −10.73 kcal/mol). Besides, P274 also became more stable to bind mismatch containing DNA (−1.41 to −2.69 kcal/mol) (Figure 4E and Supplementary Figure S5A). The binding free energy of R236 hardly changed in 274-VG-275 (−10.06 to −9.2 kcal/mol), which is also consistent with hRAD51 273-VG-274 variant. As predicted, the binding free energy of R236 to mismatched DNA increased in 274-PD-275 (−10.68 to −9.2 kcal/mol) and 274-VD-275 (−8.86 to −8.2 kcal/mol) variants, indicating a loss of R236’s stabilization to the mismatch containing DNA caused by the introduction of D275 mutation.

In conclusion, the two residues VD (hRAD51), corresponding to PG (hDMC1) in Loop2 region could affect the mismatch tolerance of the two recombinases through an interaction with the Loop1 region R235 (or R236). R235 could facilitate the complementary strand binding when encountering the fully paired DNA through π–π interaction. However, the rigid electrostatic interaction formed by R-D may prevent R235 from binding to the loose and flexible mismatch containing complementary strand. Additionally, V and P could both directly stabilize DNA during strand exchange, while P appears even stronger than V for binding mismatch containing DNA (Figure 2D; Supplementary Tables S3 and S4).

## DISCUSSION

HR of DNA repair in eukaryotic cells is mainly conducted by two recombinases, Rad51 and Dmc1. Rad51 is not only responsible for DNA double-strand break repair in mitosis, for example, repairing interrupted replication forks occurring in G2/S phase (63,64) but has also been identified during meiosis to facilitate HR mediated by Dmc1 (18,65). However, the only function of Dmc1 is to mediate HR between parental chromosome for genetic diversity in meiosis I (66). The crucial difference between Rad51 and Dmc1 is the mismatch tolerance during strand exchange, which is the central step of HR. Here, we integrated cryo-EM, MD simulation and smFRET assays to provide a comprehensive view at atomic and molecular levels to elucidate how two key residues in Loop2 region P274 and G275 of hDMC1 dominate the mismatch tolerance hDMC1. One very recent study (24) provided the high resolution of hDMC1–DNAs complexes by cryo-EM which verified the authenticity of our MD simulation of hDMC1–DNAs complexes. Furthermore, the same work also proposed a decisive role of R-D interaction for fidelity governing during strand exchange. In addition, our work suggested that, in the absence of this critical D-R interaction, a relatively weak interactions either from V or P could preserve the π–π interaction between R and mismatch-containing complementary strand.

During strand invasion, the base-flipping of the first two bases in a triplet of the complementary strand to pair with the invading strand is rapid, while flipping and pairing of the third base within that triplet is slow and the rate-limiting step (2,67). According to our simulation results, we suggested that the electrostatic interaction between D274 and R235 could be the reason to cause the slow base flipping of the third bases to slow down the speed of strand exchange and to guarantee fidelity (31,68) (Figure 4G). Furthermore, our smFRET assays underline the importance of ‘8 nt-microhomology’ required for DNA strand exchange because this process rarely occurs with 6nt-paired dsDNAs in our smFRET assay for both hRAD51 wt and hDMC1 wt.

Our smFRET assays also clearly showed, for both hRAD1 and hDMC1, that PG variants displayed higher exchange efficiencies than VG variants, supported by strand exchange efficiency of 8 nt-paired group (Figure 3E and F). We used MD to illustrate the intrinsic difference between PG and VG. In the absence of the strong interaction of R-D which dominates the mismatch tolerance during exchange, the oxygen in the main chain of P274 could form another weaker interaction with the guanidine group of R236 in hDMC1 wt (Figure 4E and Supplementary Figure S5A), and same interaction was also found between V274 to R235 in hRAD51 273-VG-274 (Supplementary Figure S5B). The distance of that O and -NH in PG variants was uniformly arranged around 2.8 Å (Supplementary Figure S5D), forming the hydrogen bond to stable R235. However, the distance of O and -NH in VG variants is not only displayed in 2.8 Å but also fluctuated around 4–6 Å, even achieving a long distance around 16 Å (Supplementary Figure S5E), indicating the unstable interaction for R235. Therefore, to examine differences of exchange efficiencies between PG and VG, we hypothesize that the stable interaction of P-R to keep R in the more suitable conformation for coupled mismatch containing dsDNAs binding, leading to higher exchange efficiency of 273-PG-274. Conversely, V-R interaction is relatively weaker and unable to keep the proper conformation of R235 for mismatch containing dsDNA binding (Supplementary Figure S5C). Furthermore, hRAD51 variant 273-PG-274 exhibited a better defined electron density of Loop2 than that of hRAD51 wt, probably due to more dsDNA captured by hRAD51 273-PG-274 to stabilize the Loop2 region in the postsynaptic complex (Supplementary Figure S3L–O). Together, our results elucidated how the efficiency and accuracy of strand exchange are tuned by the PG/VG in Loop2 of two recombinases in the absence of the critical D-R interaction. The conserved G275 among eukaryotic Dmc1 would impose little effect on DNA–protein interactions because of its negligible side chains. It is possible that if change this G to other residues, especially amino acids with large side chains, the DNA–protein interaction could be disturbed, which might cause Dmc1 less tolerant to mismatches.

It is found that Loop1 and its key residues were crucial for DNA exchange and mismatch tolerance during HR (32,69). The compound RS-1, specifically stimulating the hRAD51 DNA binding during strand exchange (36), is verified in hRAD51-ssDNA-RS-1 structure that RS-1 is located close to Loop1 of the adjacent hRAD51 in this assembled helical style rather than in single protomer binding (Figure 1C) and might facilitate the stability of Loop1 region which is very important for DNA binding (22). Recently, some further insight was obtained regarding the coordination of Loop1 and Loop2 regions to acquire mismatch tolerance (70). Moreover, our results demonstrated that the Loop1 region does not work independently, and Loop2 serves as a proofreading checkpoint during strand exchange via its interactions with Loop1 whose residues contribute more for DNA binding than those of Loop2 (Supplementary Tables S3 and S4). The tight coupling of R235 with D274 in hRAD51, corresponding to R236 with P274 in hDMC1, underlines the importance of cooperation of the two loop regions for fidelity control of these recombinases (Figure 4).

HR between different parental alleles by chromatid crossover during meiosis is mainly mediated by Dmc1, which is essential to permit heterologous DNA exchange to promote the genetic diversity after chromosomal segregation. However, the high mismatch tolerance could give rise to genome instability by permissive gross and abundant repeats (71). This dilemma is likely solved by a cooperative functional pattern between Dmc1 and Rad51. Rad51 could be an accessory factor of Dmc1 (18) for preventing accumulation of abundant recombination products and facilitating the efficient cross-over region formation mediated by Dmc1. Successful genetic recombination and inheritance requires precise regulation of the cooperation of Dmc1 and Rad51 during meiosis HR, the mechanism of which is worth more exploration and studies in the future.

## DATA AVAILABILITY

The EM reconstructions and their corresponding atomic coordinates have been deposited in the Electron Microscopy Data Bank, under accession codes EMD-31158 (hRAD51 wt presynaptic complex with RS1), EMD-31160 (hRAD51 wt postsynaptic complex), EMD-31155 (hRAD51 273-PG-274 postsynaptic complex), EMD-31153 (ScDmc1 wt presynaptic complex) and EMD-31154 (ScDmc1 wt postsynaptic complex); and in the Protein Data Bank, under accession codes PDB-7EJC (hRAD51 wt presynaptic complex with RS1), PDB-7EJE (hRAD51 wt postsynaptic complex), PDB-7EJ6 (ScDmc1 wt presynaptic complex) and PDB-7EJ7 (ScDmc1 wt postsynaptic complex). Source data files and construct materials related to this work are available upon request.

## Supplementary Material

gkab1141_Supplemental_Files
