# Supplementary material for: Mechanisms of distinctive mismatch tolerance between Rad51 and Dmc1 in homologous recombination
Source: Nucleic Acids Res. 2021 Dec 6;49(22):13135–49. doi: 10.1093/nar/gkab1141 (PMC8682777; doi:10.1093/nar/gkab1141)
Supplement: gkab1141_Supplemental_Files [file gkab1141_supplemental_files.zip › Supplementary Information -revised.docx]

**Supplementary Information for**

**Mechanisms of distinctive mismatch tolerance between Rad51 and Dmc1 in homologous recombination**

Jingfei Xu^1#^, Lingyun Zhao^1#^, Sijia Peng^1#^, Huiying Chu^1#^, Rui Liang^1^, Meng Tian^1^, Philip P. Connell^1^, Guohui Li*^1^, Chunlai Chen*^1^, Hong-Wei Wang*^1^

^#^These authors contributed equally to this work

* To whom correspondence should be addressed. Email: hongweiwang@tsinghua.edu.cn (H.-W.W.), [chunlai@mail.tsinghua.edu.cn](mailto:chunlai@mail.tsinghua.edu.cn) (C. C.), ghli@dicp.ac.cn (G.-H.L)

**This PDF file includes:**

**Figures S1 to S5**

**Tables S1 to S4**

**Legends for Videos S1 to S2**


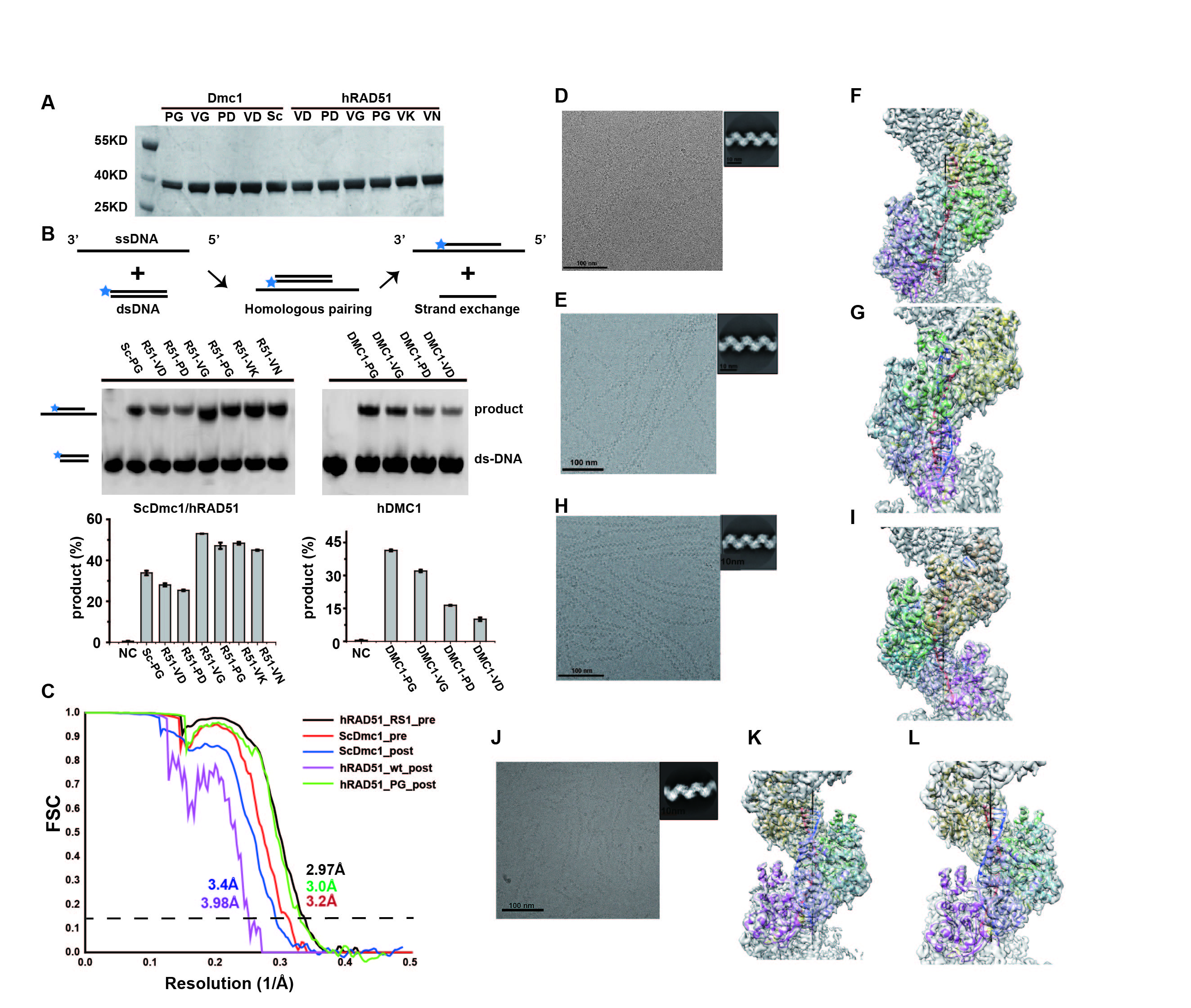


**Supplementary Figure 1. Purification and cryo-EM 3D structure reconstructions of hRAD51 and ScDmc1.**

(A) SDS-PAGE of purified wt and variants of hRAD51and hDMC1 and ScDmc1 wt (Sc in panel A).

(B) The purified variants of hRAD51, hDMC1 and ScDmc1 were analyzed by homologous DNA pairing assays. "Sc" means ScDmc1 wt, "R51" means hRAD51, "NC" means negative control without protein in assays. The error bars denote the SEM of more than 3 repeats of experiment.

(C) Gold standard FSC curves of hRAD51 presynaptic complex with small molecular RS1, ScDmc1 presynaptic complex, ScDmc1 postsynaptic complex, hRAD51 wt postsynaptic complex, and hRAD51 273-PG-274 postsynaptic complex, respectively.

(D) A representative cryo-EM micrograph and 2D average of ScDmc1 presynaptic complex (E).

(F) A representative cryo-EM micrograph and 2D average of ScDmc1 postsynaptic complex (G).

(H) A representative cryo-EM micrograph and 2D average of hRAD51-RS1 presynaptic complex (I).

(J) A representative cryo-EM micrograph and 2D average of hRAD51 273-PG-274 postsynaptic complex (K).

(L) Cryo-EM density maps of 3D reconstructions of hRAD51 wt postsynaptic complex, respectively. The representative micrographs and 2D average was published before (1).


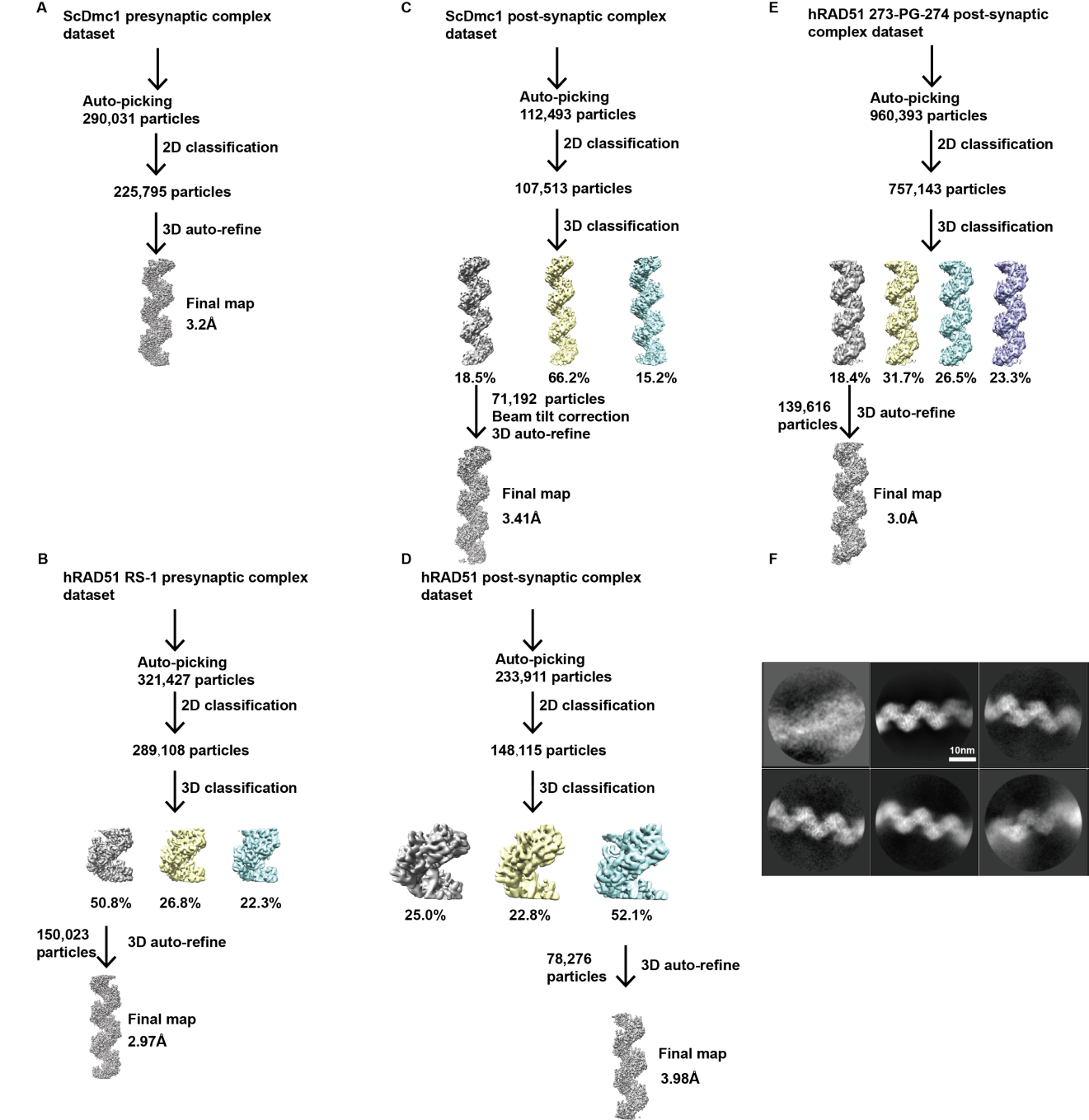


**Supplementary Figure 2. Data collection and image processing.**

(A-E) Flow-charts of the cryo-EM image processing and 3D reconstructions for the hRAD51-DNAs and ScDmc1-DNAs complexes.

(F) The examples of discarded classes of "bad particles" during helical 3D reconstructions.

**
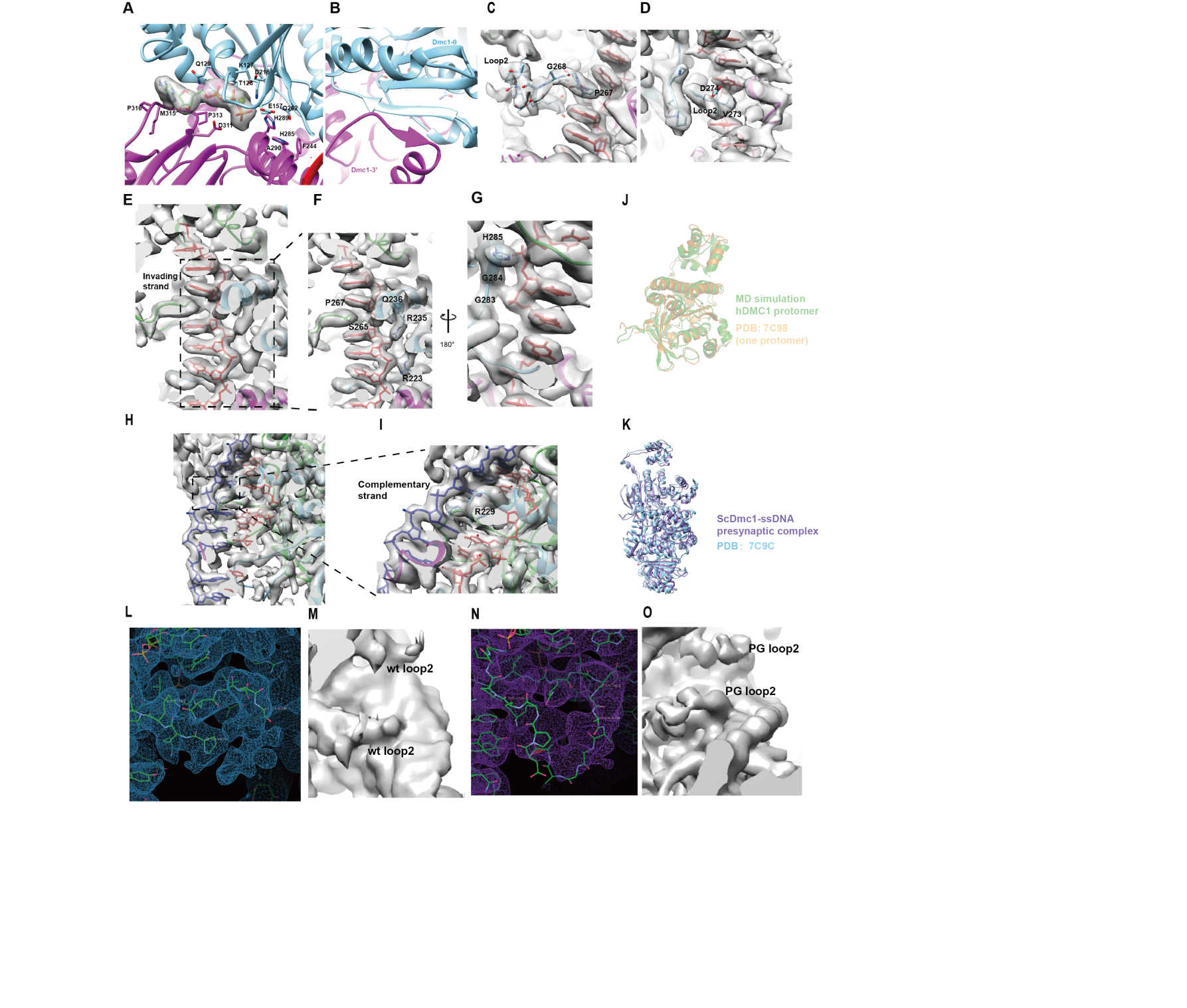
**

Supplementary Figure 3. Key structural features and density maps of ScDmc1 and hRAD51 presynaptic and postsynaptic complexes.

(A) ATP is buried in the pocket formed by two neighboring ScDmc1 protomers. Key amino acids involved in ATP binding and hydrolysis are labeled.

(B) The interaction of conserved beta strands formed at the protomer-protomer interface in ScDmc1.

(C) Intact Loop2 density map of ScDmc1 presynaptic complex.

(D) Disconnected Loop2 atomic model of the hRAD51 wt presynaptic complex with RS-1.

(E) The EM density map of invading strand ssDNA and ScDmc1 interaction in the presynaptic state. The three consecutive protomers are presented as ScDmc1-5’ (green), ScDmc1-0 (cyan) and ScDmc1-3’ (purple).

(F) A zoomed-in view of the region in (E) with the key residues labeled.

(G) An 180-degree rotation view of (F) with other key residues labeled.

(H) The EM density map of the ScDmc1 postsynaptic filament around dsDNA.

(I) A zoomed-in view of the region in (H) with R229 labeled.

(J) The superimposition of models by MD simulated hDMC1 and one protomer of hDMC1 from PDB: 7C98.

(K) The superimposition of models of ScDmc1-ssDNA presynaptic filament with hDMC-ssDNA filament of PDB: 7C9C.

(L-M) hRAD51 wt postsynaptic complex with fully homologous dsDNA shows disconnected Loop2 and its EM density.

(N-O) Intact Loop2 atomic model of hRAD51 273-PG-274 postsynaptic complex with fully homologous dsDNA and its EM density map.

**
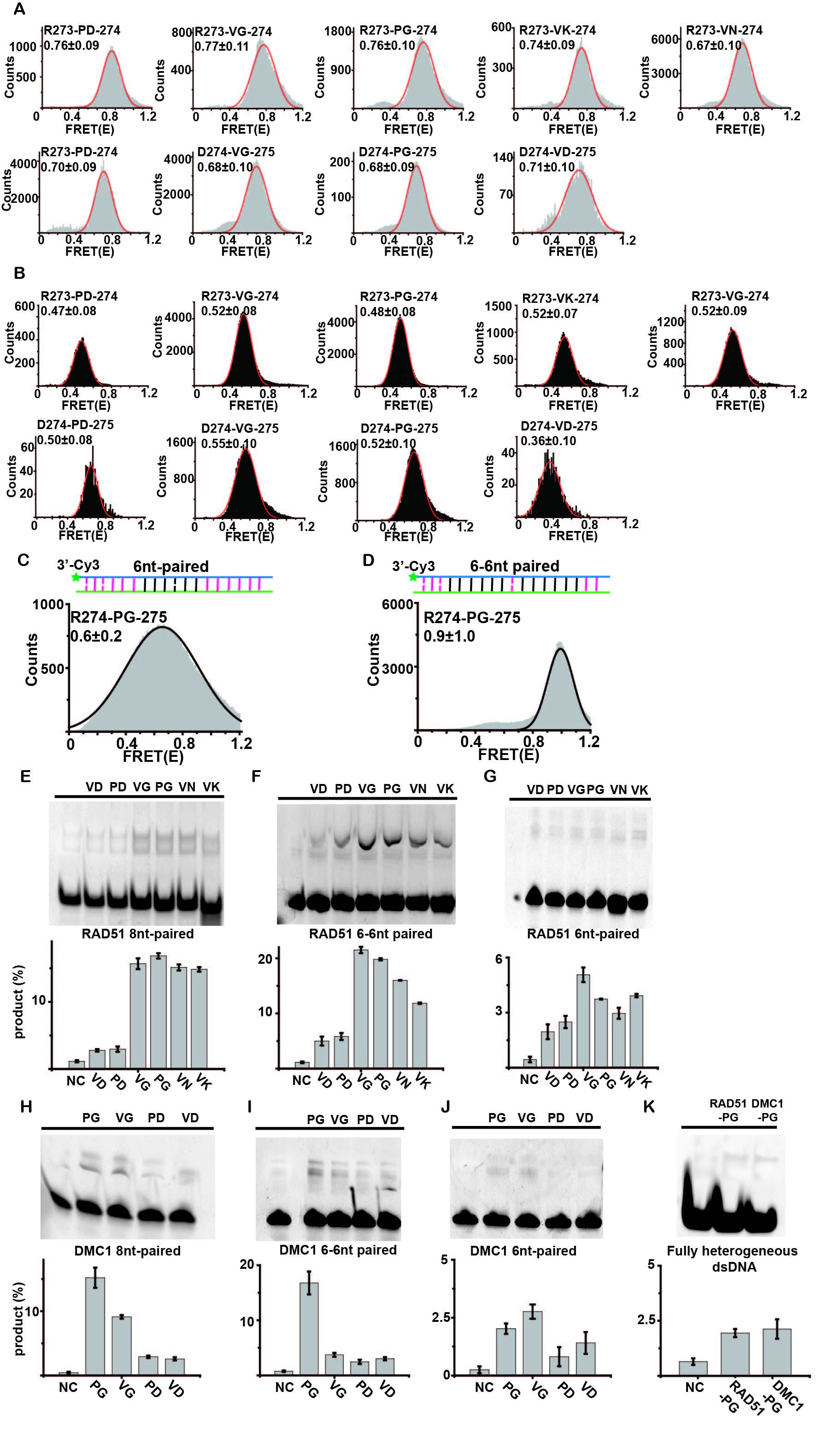
**

**Supplementary Figure 4. Distributions of smFRET efficiency between invading strand (strand I) and complementary strand (strand C) after strand exchange**

(A) smFRET assays were performed with fully-paired dsDNA.

(B) smFRET assays were performed with 8nt-paired dsDNA. R is hRAD51 and D is hDMC1. Peak center and standard deviation (sigma) of the Gaussian peaks (red curves) are listed.

(C-D) smFRET assays were performed with 6nt-paired dsDNA and individual mismatch 6-6nt paired dsDNA. Peak center and standard deviation (sigma) of the Gaussian peaks (black curves) are listed.

(E-K) The strand exchange assays were performed with 8nt-paired, 6-6nt paired, and 6nt-paired dsDNA. NC stands for negative control without protein in assay. The error bars denote the SEM of more than 3 repeats of experiment.


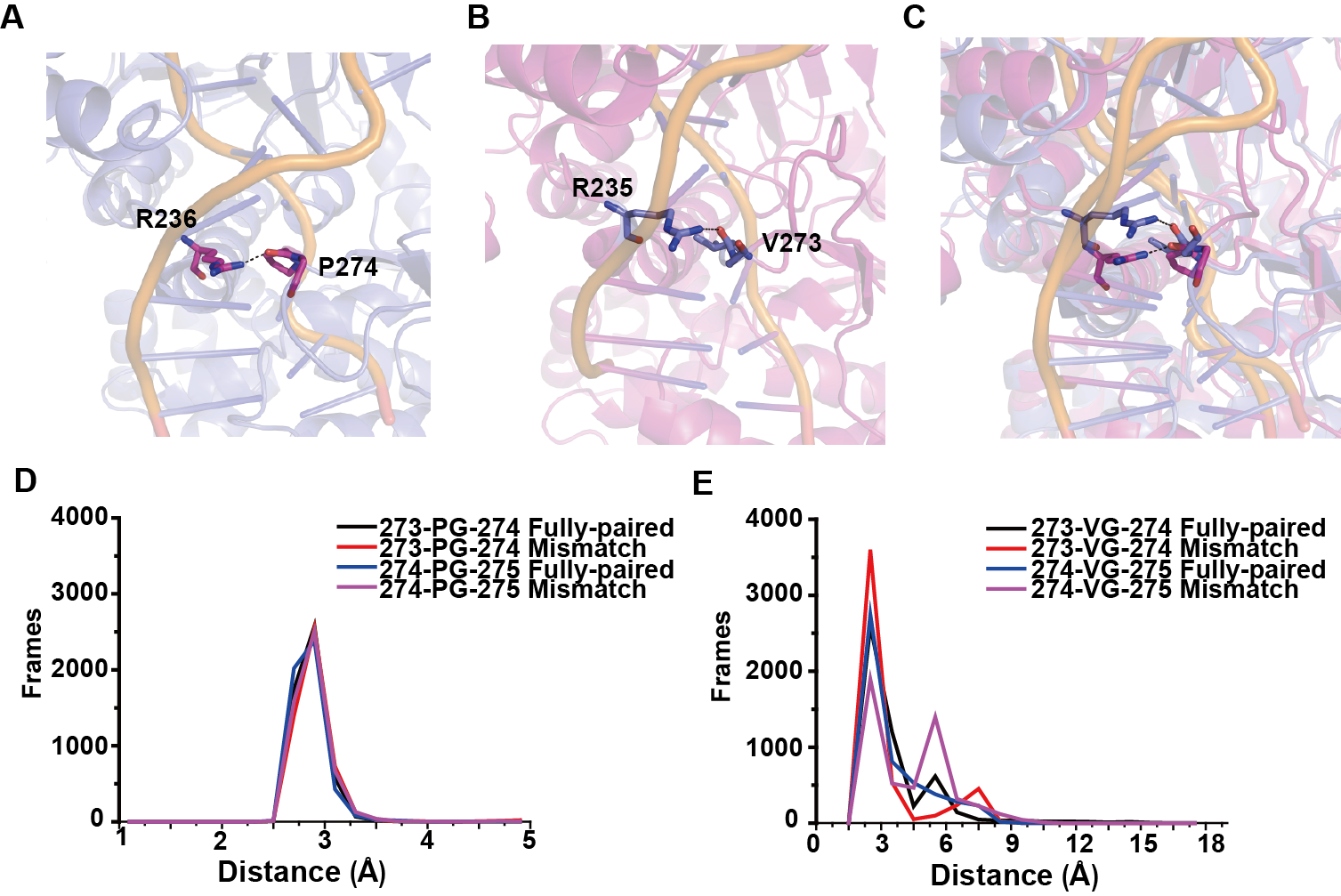


**Supplementary Figure 5. The mechanism of difference of mismatch tolerance of PG and VG variants.**

(A) A snapshot of the MD simulation to show the possible interaction between O of P274 and -NH of R236 for mismatch-containing DNA binding of hDMC1 wt which is similar to other PG variants (related to Figure 4E-F).

(B) A snapshot of the MD simulation to show the possible interaction between O of V273 and -NH of R235 for mismatch-containing DNA binding of hRAD51 273-VG-274 variant which is similar to hDMC1 274-VG-275.

(C) The alignment of MD simulation snapshots of PG and VG to show the conformational difference of argenine for mismatch-containing dsDNA binding.

(D) The distributions of distance between -NH and O in PG variants, corresponding to panel (A). The y-axis is number of frames and the total number is 5000 frames lasting for 1μs.

(E) The distributions of distance between -NH and O in VG variants, corresponding to panel (B).

**Supplementary Table S1.** **Oligonucleotides used in this work**

| Oligos | Sequence |
| --- | --- |
| Oligo 1 | 5'-TAAATAAGATAAGGATAATACAAAATAAGTAAATGAATAAACAGAGAAAATAAAGTAAAGGATATAAAAAATGAACATAAAGAATAAGTAAATGAATAA-3’ |
| Oligo 2 | 5'-Cy5-CTTTATTTTCTCTGTTTATTCATTTACTTATTTTGTATTA-3' |
| Oligo 3 | 5'-TAATACAAAATAAGTAAATGAATAAACAGAGAAAATAAAG-3' |
| Oligo 4 | 5'-TAAATAAGATAAGGATAATACAAAATAAGTAAATGAATAAACAGAGAAAATAAAGTAAAGGATATAAAAAATGAACATAAAGAATAAGTAAATGAATAAAACATAATAGGAATAAATATAGGAAATGAAATAAAAGAGACATAAATAAGA-3' |
| Oligo 5 | 5'-TCTTATTTATGTCTCTTTTATTTCATTTCCTATATTTATTCCTATTATGTTTTATTCATTTACTTATTCTTTATGTTCATTTTTTATATCCTTTACTTTATTTTCTCTGTTTATTCATTTACTTATTTTGTATTATCCTTATCTTATTTA-3' |
| Oligo 6 | 5'-Cy5-CCAATGTAGAGACTAT-3' |
| Oligo 7 | 5’-biotin-ATAGTCTCTACATTGG CTAGCTG AAGATTATATTTAAACCT-3’ |
| Oligo 8 | 5'- AGGTTTAAATATAATCTT -Cy3-3' |
| Oligo 9 | 5'- AAGATTATATTTAAACCT -3’ |
| Oligo 10 | 5'-TCCGCCAAATATAACTCC-Cy3-3' |
| Oligo 11 | 5'-GGAGTTATATTTGGCGGA-3' |
| Oligo 12 | 5'-TCCGCCTAATATAGCTCC-Cy3-3' |
| Oligo 13 | 5'-GGAGCTATATTAGGCGGA-3' |
| Oligo 14 | 5'-biotin-ATAGTCTCTACATTGG CTAAGCTGAAGATTATAT-3' |
| Oligo 15 | 5'-CGATAATCATCAGCTGCT-3' |
| Oligo 16 | 5'-AGCAGCTGATGATTATCG-3' |
| Oligo 17 | 5'-TTTTTTTTT-3' |
| Oligo 18 | 5'-AAAAAAAAA-3' |
| Oligo 19 | 5'-AAAAGAAAA-3' |

**Supplementary Table S2. Cryo-EM statistics and model refinement for ScDmc1 and hRAD51 presynaptic and postsynaptic complexes.**

|  | ScDmc1 presynaptic | ScDmc1 postsynaptic | | hRAD51 RS-1 presynaptic | | hRAD51 postsynaptic | hRAD51  273-PG-274 postsynaptic |
| --- | --- | --- | --- | --- | --- | --- | --- |
| PDB ID | 7EJ6 | 7EJ7 | | 7EJC | | 7EJE | --- |
| EMDB ID | 31153 | 31154 | | 31158 | | 31160 | 31155 |
| Data collection and processing | | | | | | |  |
| Microscope | Titan Krios with Cs-corrector | Titan Krios | | Titan Krios with Cs-corrector | | Titan Krios | Titan Krios |
| Detector | Falcon II | Gatan K2 Summit | | Falcon II | | Gatan K2 Summit | Gatan K2 Summit |
| Pixel size (Å) | 0.885 | 1.025 | | 0.885 | | 1.306 | 1.0825 |
| Total electron dose (e-/Å^2^) | 48 | 48 | | 48 | | 50 | 40 |
| Exposure rate (e^-^/pixel/sec) | 20.9 | 9.6 | | 20.9 | | 10.6 |  |
| Defocus range (µm) | -1.0 to -3.0 | -1.0 to -3.5 | | -1.0 to -3.0 | | −1.5 to −2.5 | −1.5 to −2.5 |
| Micrographs collected | 2175 | 1502 | | 1159 | | 528 | 2082 |
| Micrographs used | 2154 | 1333 | | 1115 | | 484 | 2055 |
| Reconstruction  Software | Relion | | | | | |  |
| Total extracted particles | 290,031 | 112,493 | | 321,427 | | 233,911 | 960,393 |
| Number of particles used for final refinement | 225,795 | 71,192 | | 150,023 | | 78,276 | 139,616 |
| Symmetry | Helical | | | | | |  |
| Resolution(Å)  0.143 after post-processing | 3.21 | | 3.41 | | 2.97 | 3.98 | 3.0 |
| Refinement | | | | | | |  |
| Software | Phenix.real_space_refine in PHENIX software package | | | | | |  |
| Resolution (Å) | 3.20 | 3.40 | | 3.0 | | 3.98 | --- |
| Model composition | | | | | | |  |
| Number of protein atoms | 7308 | 7308 | | 7329 | | 7329 |  |
| Number of nucleic acids atoms | 177 | 366 | | 177 | | 366 |  |
| Number of ligand atoms | 96 | 96 | | 96 | | 96 |  |
| R.M.S deviations  Bonds lengths (Å) | 0.004 | 0.004 | | 0.006 | | 0.008 |  |
| Bonds angles (°) | 0.629 | 0.635 | | 0.634 | | 1.243 |  |
| Validation | | | | | | |  |
| MolProbity overall score | 2.07 | 2.07 | | 2.05 | | 2.31 |  |
| All-atom clashscore | 10.83 | 9.97 | | 7.27 | | 7.63 |  |
| Rotamer outliers (%) | 0 | 0 | | 2.42 | | 2.28 |  |
| C-beta deviations | 0 | 0 | | 0 | | 11 |  |
| Ramachandran plot statistics  Preferred (%) | 90.98 | 90.13 | | 95.10 | | 86.83 |  |
| Allowed (%) | 8.39 | 9.24 | | 4.90 | | 11.76 |  |
| Outlier (%) | 0.64 | 0.64 | | 0 | | 1.41 |  |

**Supplementary Table S3. Binding free energies of different residues for hRAD51-dsDNA complexes**

| **273-VD-274(wt)** | | | | **273-PD-274** | | | | **273-VG-274** | | | | **273-PG-274** | | | |
| --- | --- | --- | --- | --- | --- | --- | --- | --- | --- | --- | --- | --- | --- | --- | --- |
| **Fully-paired** | | **Mismatch** | | **Fully-paired** | | **Mismatch** | | **Fully-paired** | | **Mismatch** | | **Fully-paired** | | **Mismatch** | |
| **Residue** | **Energy** | **Residue** | **Energy** | **Residue** | **Energy** | **Residue** | **Energy** | **Residue** | **Energy** | **Residue** | **Energy** | **Residue** | **Energy** | **Residue** | **Energy** |
| C:R235(844) | -10.81 | **B:R235(529)** | **-9.46** | **B:R235(529)** | **-10.90** | **B:R235(529)** | **-10.04** | C:R235(844) | -9.32 | C:R235(844) | -9.91 | **B:R235(529)** | **-10.03** | **B:R235(529)** | **-10.09** |
| **B:R235(529)** | **-10.82** | C:R235(844) | -9.59 | C:R235(844) | -11.08 | C:R235(844) | -9.04 | **B:R235(529)** | **-8.74** | **B:R235(529)** | **-8.28** | C:R235(844) | -9.93 | C:R235(844) | -9.01 |
| **B:V273(567)** | **-2.71** | **B:V273(567)** | **-1.31** | **A:P273(252)** | **-2.42** | C:R229(838) | -1.18 | **A:V273(252)** | **-1.02** | A:R235(214) | -4.32 | A:R235(214) | -3.25 | A:R235(214) | -2.70 |
| A:R235(214) | -2.12 | A:R235(214) | -3.92 | **B:P273(567)** | **-2.87** | **B:P273(567)** | **-1.27** | A:R235(214) | -5.05 | **B:V273(567)** | **-1.84** | **A:P273(252)** | **-1.90** | **A:P273(252)** | **-1.55** |
| B:G234(530) | -1.93 | B:G234(530) | -3.70 | A:R235(214) | -3.24 | **A:P273(252)** | **-1.96** | B:P271(567) | -2.13 | **A:V273(252)** | **-1.18** | B:G234(530) | -1.83 | **B:P273(567)** | **-2.14** |
| **A:V273(252)** | **-1.94** | B:Y232(528) | -2.87 | B:G234(530) | -1.92 | A:R235(214) | -3.66 | C:Y232(845) | -1.71 | C:Y232(845) | -1.86 | B:Q272(568) | -1.50 | C:R229(838) | -2.01 |
| C:Y232(845) | -1.81 | C:Y54(667) | -1.12 | C:Y232(845) | -1.78 | B:G234(530) | -2.06 | B:G234(530) | -1.70 | B:G234(530) | -1.60 | C:Y232(845) | -1.41 | B:G234(530) | -1.37 |
| C:Y54(667) | -1.11 |  |  |  |  | C:Q268(881) | -1.24 |  |  | B:Q272(568) | -1.56 | C:V269(882) | -1.39 | C:Y232(845) | -1.30 |
|  |  |  |  |  |  | C:Y232(845) | -1.92 |  |  | C:V269(882) | -1.30 | A:G274(253) | -1.20 | B:G236(532) | -1.01 |
|  |  |  |  |  |  |  |  |  |  | B:G275(569) | -1.29 |  |  |  |  |
|  |  |  |  |  |  |  |  |  |  |  |  |  |  |  |  |
|  |  |  |  |  |  |  |  |  |  |  |  |  |  |  |  |

| **273-VK-274** | | | | **273-VN-274** | | | |
| --- | --- | --- | --- | --- | --- | --- | --- |
| **Fully-paired** | | **Mismatch** | | **Fully-paired** | | **Mismatch** | |
| **Residue** | **Energy** | **Residue** | **Energy** | **Residue** | **Energy** | **Residue** | **Energy** |
| C:R235(844) | -10.28 | C:R235(844) | -9.02 | C:R235(844) | -9.248 | C:R235(844) | -10.05 |
| **B:R235(529)** | **-8.67** | **B:R235(529)** | **-8.6** | **B:R235(529)** | **-8.56** | **B:R235(529)** | **-9.09** |
| A:K274(253) | -3.06 | A:R235(214) | -3.95 | A:R235(214) | -3.416 | A:R235(214) | -3.02 |
| **B:V273(567)** | **-2.29** | C:K274(883) | -2.32 | B:G234(530) | -1.888 | B:Q272(568) | -2.502 |
| A:R235(214) | -2.16 | **B:V273(567)** | **-2.25** | C:Y232(845) | -1.79 | **B:V273(567)** | **-1.962** |
| C:Y232(845) | -1.68 | B:Q272(568) | -1.78 | **B:V273(567)** | **-1.416** | C:Y232(845) | -1.9 |
| B:G234(530) | -1.60 | C:Y232(845) | -1.67 | B:A238(534) | -1.334 | B:G234(530) | -1.48 |
| B:L236(532) | -1.59 | B:G234(530) | -1.51 |  |  | B:G236(532) | -1.3 |
| B:Q272(568) | -1.48 | A:K274(253) | -1.34 |  |  | B:A238(534) | -1.21 |
| A:P273(252) | -1.28 | B:G236(532) | -1.1 |  |  |  |  |

The complementary strand is used as a ligand. A, B, and C stand for different protomers. The protomer B is the nearest to DNA mismatch region among three protomers, related to Figure. 4H-J. Energy is in kcal/mol.

The important residues related to this study are displayed in bold font.

**Supplementary Table S4. Binding free energies of different residues for hDMC1-dsDNA complexes**

| **274-PG-275(wt)** | | | | **274-VG-275** | | | | **274-PD-275** | | | | **274-VD-275** | | | |
| --- | --- | --- | --- | --- | --- | --- | --- | --- | --- | --- | --- | --- | --- | --- | --- |
| **Fully-paired** | | **Mismatch** | | **Fully-paired** | | **Mismatch** | | **Fully-paired** | | **Mismatch** | | **Fully-paired** | | **Mismatch** | |
| **Residue** | **Energy** | **Residue** | **Energy** | **Residue** | **Energy** | **Residue** | **Energy** | **Residue** | **Energy** | **Residue** | **Energy** | **Residue** | **Energy** | **Residue** | **Energy** |
| C:R236(850) | -5.43 | **B:R236(533)** | **-10.73** | **B:R236(533)** | **-10.06** | **B:R236(533)** | **-9.2** | **B:R236(533)** | **-10.68** | C:R236(850) | -9.76 | C:R236(850) | -8.90 | C:R236(850) | -9.64 |
| **B:R236(533)** | **-6.59** | C:R236(850) | -11.24 | C:R236(850) | -8.30 | C:R236(850) | -8.67 | C:R236(850) | -9.16 | **B:R236(533)** | **-9.20** | **B:R236(533）** | **-8.86** | **B:R236(533)** | **-8.20** |
| **A:P274(254)** | **-1.41** | **A:P274(254)** | **-2.69** | A:R236(216) | -2.83 | A:R236(216) | -4.96 | A:R236(216) | -5.08 | B:A272(571) | -1.81 | A:R236(216) | -4.12 | A:R236(216) | -6.09 |
| C:G235(853) | -3.12 | C:M53(671) | -1.08 | C:M53(671) | -2.62 | B:A272(571） | -1.20 | B:A272(571) | -1.47 | A:R236(216) | -2.70 | C:M53(671) | -3.36 | B:A272(571) | -1.60 |
| A:R236(216) | -3.45 | B:E238(537) | -1.33 | B:G235(534) | -1.99 | C:M53(671) | -2.31 | B:A240(537) | -1.01 | **A:P274(254)** | **-1.05** | B:G235(534) | -2.27 | C:M53(671) | -2.75 |
| B:D273(572) | -2.68 | B:A272(571) | -2.56 | **A:V274(254)** | **-1.66** | B:E238(537) | -1.52 | C:M53(671) | -2.06 | B:E238(537) | -1.04 | C:F233(851) | -1.53 | B:G235(534) | -2.38 |
| **B:P274(573)** | **-2.24** | B:G235(534) | -1.47 | B:A272(571) | -1.12 | B:G235(534) | -2.31 | **A:P274(254)** | **-1.20** | B:G235(534) | -2.36 | B:A272(571） | -1.62 | C:F233(851) | -2.17 |
| B:G235(534) | -1.99 | A:R236(216) | -4.26 | B:A240(537) | -1.22 | C:F233(851) | -1.67 | B:G235(534) | -2.18 | C:F233(851) | -2.44 |  |  |  |  |
| C:F233(851) | -1.77 | C:F233(851) | -2.79 | C:F233(851) | -1.93 | B:A276(575) | -1.39 | C:F233(851) | -1.78 |  |  |  |  |  |  |
| C:P274(892) | -1.69 |  |  |  |  |  |  | A:A276(256) | -1.08 |  |  |  |  |  |  |
| C:R236(854) | -1.51 |  |  |  |  |  |  | B:E238(537) | -1.01 |  |  |  |  |  |  |
| C:A272(890) | -1.06 |  |  |  |  |  |  |  |  |  |  |  |  |  |  |

The complementary strand is used as a ligand. A, B, and C stand for different protomers.

Binding free energies between DNA and all three protomers were calculated and listed here. The protomer B is the nearest to mismatch DNA region (Figure. 4H-J), whose values were used in the maintext to compare the contribution of different residues to stabilize mismatches.

Energy is in kcal/mol.

The important residues related to this study are displayed in bold font.

Supplementary Video S1. Structure comparison of Loop2 region in hRAD51 and hDMC1.

Supplementary Video S2. MD simulation of interaction D274 and R235 in hRAD51. The simulation captured the dynamic interaction of D274 and R235 in the hRAD51 postsynaptic complex. The electrostatic interaction of D274 and R235 restricted dsDNA binding. Meanwhile, R235 interacts with two bases from neighboring triplets through a π-π interaction. Red ssDNA stands for invading strand, and blue ssDNA stands for complementary strand. Three hRAD51 protomers are in green, sky blue, and magenta from 5'-3'. The two-base group of nucleotides is shown in red pentagons.

References

1. Xu J*, et al.* (2017) Cryo-EM structures of human RAD51 recombinase filaments during catalysis of DNA-strand exchange. *Nat Struct Mol Biol* 24(1):40-46.
